# Criteria for timely euthanasia of compromised pigs based on expert opinion and longitudinal data collection on farm

**DOI:** 10.1186/s40813-026-00532-2

**Published:** 2026-08-08

**Authors:** Julia D. Kschonek, Maria Hartmann, Kathrin Deters, Annika Joost, Moana Miller, Jennifer Reinmold, Henrike Maiti, Isabel Hennig-Pauka, Nicole Kemper, Christin Kleinsorgen, Karl-Heinz Tölle, Lothar Kreienbrock, Michael Wendt, Elisabeth Grosse Beilage

**Affiliations:** 1 Institute of Biometry, Epidemiology and Information Processing (IBEI), WHO-CC for Health at the Human-Animal-Environment Interface, University of Veterinary Medicine, Foundation, Bünteweg 2, 30559 Hannover, Germany; 2 Field Station for Epidemiology, University of Veterinary Medicine, Foundation, Büscheler Str. 9, 49456 Bakum, Hannover, Germany; 3 Institute for Animal Hygiene, Animal Welfare and Farm Animal Behavior, University of Veterinary Medicine, Foundation, Bischofsholer Damm 15, 30173 Hannover, Germany; 4 Centre for E-Learning, Didactics, Educational Research [ZELDA], University of Veterinary Medicine, Foundation, Bünteweg 2, 30559 Hannover, Germany; 5 ISN-Projekt GmbH, Kirchplatz 2, 49401 Damme, Germany; 6 Clinic for Swine, Small Ruminants, Forensic Medicine and Ambulatory Service, University of Veterinary Medicine, Foundation, Bischofsholer Damm 15, 30173 Hannover, Germany

**Keywords:** Mercy killing, Lameness, Time frame, Course of disease, Animal welfare, Hospital pen

## Abstract

Timely euthanasia is essential for safeguarding pig welfare by preventing unnecessary pain and suffering. However, the definition of what constitutes “timely” euthanasia and which clinical signs allow identification of this time point require empirical validation. This study presents the results of an expert evaluation conducted during a continuing education course. Seventy pig-experienced veterinarians and farmers assessed on-farm recorded clinical signs, as well as photographic and video material from 130 pigs documenting the progression of disease over several days until recovery or euthanasia. For each observation day presented, participants voted for or against euthanasia and provided the reasoning underlying their decision. In total, more than 22,000 individual evaluations were collected. The time point considered appropriate for euthanasia and the associated decision criteria were analyzed and compared with the clinical signs recorded on farm. Qualitative analysis showed that expert consensus on timely euthanasia is primarily based on assessing the chance of treatment success, establishing a specific diagnosis, and evaluating clinical signs through repeated assessments. Furthermore, results indicated that euthanasia decision-making should occur within a time frame encompassing up to three re-visitations.

**Supplementary Information:**

The online version contains supplementary material available at 10.1186/s40813-026-00532-2.

## Background

The health assessment of individual pigs and the herd as a whole is part of the daily work of veterinarians and farmers. When pigs are diseased or injured, this assessment inevitably requires a decision between (further) treatment and euthanasia. If a compromised pig is incurably diseased, the owner or animal caretaker must ensure that the animal does not experience unnecessary pain or suffering [5]. If such a condition cannot be alleviated through treatment and recovery is unlikely, the pig must be euthanized. In this context, recognizing an incurable disease or injury at the earliest possible stage is crucial for ensuring timely euthanasia decisions and actions.

Several studies indicate that the appropriate time point for euthanasia is not always identified or implemented in practice [6, 17]. Consequently, guidelines have been developed to support timely euthanasia decision-making [1, 12]. However, these guidelines often omit a clear definition of what constitutes “timely”, and the clinical indicators of incurable health states are rarely supported by empirical evidence.

To address this issue, Mullins et al., [13] investigated expert opinions regarding the timing of pig euthanasia. Experts were presented with selected disorders and clinical signs and asked to assign them to predefined time frames considered appropriate for euthanasia. However, the study did not examine whether these conditions posed particular challenges for timely decision-making, whether the range of clinical signs reflected real-world case complexity, or how changes in clinical status over time influenced expert assessments. Furthermore, Mullins et al., [13] reported that euthanasia guidelines were often not followed and that considerable variability existed among expert opinions.

Thus, a knowledge gap remains regarding which clinical signs pig veterinarians and farmers consider relevant when evaluating real cases and disease progression without predefined time frames. In particular, a shared perspective on the clinical signs presented at the time of timely euthanasia and the reasons underlying such decisions is lacking.

Accordingly, this study addresses the research question: which clinical signs characterize pigs on the day of euthanasia when the majority of pig experts consider the decision to be timely? To answer this question, expert evaluations were conducted for 130 pigs across several days, documenting the progression of their disease until either recovery or euthanasia. Based on these expert assessments and the corresponding clinical observations, criteria for timely euthanasia of compromised pigs are derived and discussed, with the aim of supporting veterinarians and farmers in making timely euthanasia decisions in practice.

## Methods

### Empirical data set

The evaluation dataset was derived from a larger dataset comprising 3,272 clinical examinations collected over a period of one and a half years (November 2022 to April 2024). These examinations documented general health assessments, examinations of specific disease conditions or organ systems, and treatment-related data, encompassing 318 potential entry variables. Data were collected by three project veterinarians (“examiners”), who visited a total of five commercial pig farms on average every second to third day. Four farms were closed systems and one farm was a mixed fattening and piglet rearing system. On days without project visits, compromised pigs were managed by farmers, caretakers, and the herd-attending veterinarians. All persons involved were informed about the project and its objectives.

In total, 615 diseased or injured pigs were included in the project. These animals were examined by project veterinarians an average of 4.2 times over a mean observation period of 10.9 days, until the pigs left the project either due to recovery (i.e., being considered healthy enough for continued fattening, slaughter, or early slaughter) or following a decision to euthanize. During the observation period, all pigs received treatment (including pain medication, antimicrobial and / or anti-inflammatory medication depending on clinical signs and suspected diagnoses) according to the instructions of the herd-attending veterinarians.

Most clinical examinations in the empirical dataset involved weaner pigs (42.9%), followed by fattening pigs (33.2%), suckling pigs (23.2%), and sows (0.7%). The most frequent primary diagnosis (“PD”) was lameness (60.7%), followed by the PDs tail lesions (8.8%), skin disorders (4.8%), and skin injuries (4.2%).

A similar distribution was observed among the euthanized pigs (80 out of 615 animals). These pigs accounted for 12.8% of all clinical examinations (420 examinations). On average, euthanized pigs remained in the project for 11.5 days and were examined by project veterinarians 4.2 times before a final decision for euthanasia or recovery was made. Most euthanized pigs were weaners (44.3%), followed by fattening pigs (33.8%) and suckling pigs (21.9%). In the majority of cases, the primary diagnosis among euthanized pigs was lameness (58.6%), followed by the PDs tail injuries (7.1%) and respiratory tract disorders (5.2%).

If the primary diagnosis of a pig changed during the observation period, the diagnosis recorded at the final examination day was used for categorization. Detailed data tables are provided in the Appendix (“A: Original dataset”).

### Evaluation data set

To generate a representative set of pig cases for evaluation by pig experts, several selection criteria were defined. The selected cases were required to reflect the distribution and combinations of primary diagnoses, disorders considered particularly challenging for euthanasia decision-making (e.g., low-viability pigs; [15]), and the different age classes of pigs. Based on these criteria, 51 euthanized and 79 recovered pigs were included. Three of the recovered pigs were presented twice, resulting in a total of 133 pig cases in the evaluation dataset.

Due to time constraints for the evaluators (limited to 12 h), the documented disease progression of the selected cases had to be reduced to the most representative examination days. The identification of “representative” days was determined on a case-by-case basis. In general, the first and last examination days of each empirical case were included, and additional days were selected only when the clinical examination could be supported by photo or video footage of at least medium quality. Furthermore, the number of examination days was adjusted depending on the development of the disorder, for example whether the condition progressed steadily towards euthanasia or showed fluctuating clinical signs.

Based on these criteria, three veterinarians (independent of the original examiners) selected and prepared the cases for evaluation. The clinical examinations were condensed to a total of 376 examination days, referred to in this article as “examination day presentations.”

Within the evaluation dataset, most selected pigs were weaners (42.9%), followed by fattening pigs (33.2%) and suckling pigs (23.2%). The most frequent primary diagnoses represented disorders of the locomotor system (51.3%), followed by tail-biting lesions (7.5%), central nervous system disorders (6.9%), and retarded development compared with pen mates (4.5%). On average, pigs remained in the project for 7.6 days and were examined 3.1 times.

Among the 51 euthanized pigs, the average observation period was 6.5 days, with an average of 2.8 examinations per animal. Most euthanized pigs were weaners (44.3%), followed by fattening pigs (33.8%) and suckling pigs (21.9%). The most common primary diagnoses among euthanized pigs were locomotor disorders (65.1%), followed by tail lesions (9.9%), respiratory disorders (6.6%), umbilical outpouchings (5.9%), and retarded development compared with pen mates (3.3%). Detailed tables describing the evaluation dataset are provided in the Appendix (“B: Evaluation dataset”).

### Preparation of evaluation

The evaluation was conducted as part of a continuing education course for veterinarians and farmers. The course was announced through relevant professional associations and websites. Participants were eligible to enroll if they had at least one year of experience working with pigs and were involved in, or responsible for, euthanasia decision-making and its implementation. These criteria were assessed during enrolment and verified when demographic information was collected during the evaluation.

The evaluation was organized in three phases over a period of two months (October–November 2024). In the first phase, an eight-hour hybrid meeting was held to introduce participants to the topic and to establish a shared understanding of current developments in the field as well as the concept of euthanasia. In this study, euthanasia is defined as the timely killing of a pig when no other viable option exists to reduce pain and suffering to an acceptable minimum [5]. Accordingly, euthanasia is distinct from slaughter, defined as the killing of animals for food production, and from culling, which refers to the killing of animals for purposes such as disease control within a population.

Euthanasia may be performed by either farmers or veterinarians using appropriate and legally permitted methods. These include captive bolt stunning, electrical stunning, CO₂ stunning, administration of pharmaceutical agents, and / or a combination of these methods followed by exsanguination.

The second phase consisted of a 28-day remote evaluation period, during which participants assessed the pig cases. The estimated time required for this phase was approximately 12 h. Participants received individualized access to the evaluation platform and were able to start, pause, and continue the evaluation at their convenience. They were instructed not to discuss the cases with other participants during this phase.

In the third phase, the evaluation results were presented and selected cases were discussed during a second eight-hour hybrid meeting, particularly where interpretations or decisions differed among participants. Upon completion of all evaluation phases, participants received continuing education credits.

The case evaluations, including both clinical records and video material, were administered using LimeSurvey (version 3.28.76), and data analysis was conducted in SAS^®^ (version 9.4M7, SAS Institute Inc., Cary, North Carolina, USA). Before accessing the cases, participants received a general description of the evaluation procedure, the research question and interest of the study and were required to consent to the use of their anonymized participation data for research purposes. Only after providing consent were the cases made accessible. The process of secure and ethical data collection and analysis was approved by the official data protection officer of the involved institutions.

Each case presentation was limited to a single clinical examination day for one pig and was displayed on a single webpage. Evaluators were not informed how many examination days would be presented for a given pig and therefore did not know whether a follow-up assessment would occur. Likewise, they were unaware of the empirical outcome of the case (i.e., whether the pig ultimately recovered or was euthanized in the project).

For each examination day presentation, participants were provided with a summary of the current clinical signs, the duration of the pig’s being treated in the project, and treatment records summarizing medication categories administered since the first examination day (pain medication, antimicrobial or anti-inflammatory medication). In addition, photographic and / or video material illustrating the clinical signs were provided. After reviewing the case information, participants were asked to record their decision (for or against euthanasia) and to provide the reasons underlying their assessment. An example of an original case presentation is provided in the Appendix (Appendix C: Conduct of Evaluation / Original Case Presentation).

### Data aggregation and analysis

Following each presentation of a clinical examination day, evaluators were asked how they would decide regarding euthanasia or alternative options for treatment or care (the exact questions are summarized in the Appendix, “C: Conduct of Evaluation – Overview of Questions in English”). Responses to this first question were aggregated into two categories: decisions **in favor of euthanasia** and decisions **against euthanasia** (i.e., any other decision including early slaughter).

If evaluators voted in favor of euthanasia, they were asked a follow-up question indicating whether they considered the timing of euthanasia for the specific pig and examination day to be **timely** or **late** (“should have been done earlier”). Based on these two responses, the presented pig cases were categorized into three groups.

Cases were defined as **qualified timely euthanized pigs (QE)** if more than 65% of participants decided in favor of euthanasia in the first question and more than 50% indicated in the follow-up question that the timing of euthanasia was appropriate. Cases in which participants voted against euthanasia at every examination day with a proportion exceeding 65% were classified as **qualified recovered pigs (QR)**. All remaining cases were categorized as **unclear cases (UC)**. Throughout this article, the term **“majority vote”** refers to decisions exceeding 65% agreement and meeting the criteria for either the QE or QR group. All other aggregated responses are referred to as **“general votes”.**

As a third step in the evaluation, experts were asked, using a multiple-choice format, to indicate the reasons underlying their decision for or against euthanasia. The predefined response options were: one specific symptom, a combination of symptoms, duration of disease, overall health state, severity of symptoms, or chance of treatment success. In the Results section, responses to this question are reported as relative frequencies. Statistical analyses were conducted to examine potential differences in reported reasons according to primary diagnosis and professional background of the evaluators. All results were rounded to one decimal place; therefore, totals may deviate slightly from 100%.

Depending on the selected reason(s) for euthanasia, participants were asked follow-up questions to further specify their reasoning. In most cases, these responses were provided in free-text format. Given the focus on timely euthanasia of this article, responses from participants who voted for timely euthanasia in QE cases were categorized, quantified, and reported as relative frequencies. As the number of responses depended both on the selected reason (third-step question) and on the content of the free-text response (e.g., a combination of symptoms could result in multiple coded entries), these responses are referred to as “votes” in the Results section.

In addition to reporting relevant clinical signs and decision criteria identified by evaluators, statistical analyses were conducted to compare the **evaluation dataset** with the **overall empirical dataset**. These analyses examined differences among pig categories (qualified timely euthanized [QE], qualified recovered [QR], and unclear cases [UC]) and assessed whether the clinical signs recorded on the empirical day of euthanasia corresponded with those observed on the examination day classified as QE.

Summarizing, the overall research question, which clinical signs characterize pigs on the day of euthanasia when the majority of pig experts consider the decision to be timely, was further specified (Table 1).

**Table 1:** Overview of research questions and statistical methods

Research question	Survey questions / evaluation dataset	Statistical method / test
Which clinical signs characterize pigs on the day of euthanasia when the majority of pig experts consider the decision to be timely?	**1st step** Reasons selected by experts *(3rd survey question*,* single choice)*	Categorization, descriptive statistics à relative frequencies
	**2nd step** Clinical aspects reported by participants in detail *(follow up questions to the 3rd survey question*,* free text)*	Qualitative coding, categorization, quantification, descriptive statistics à relative frequencies
	**3rd step** Clinical signs at the day of decision making comparing case groups *(evaluation data set)*	à descriptive and inferential statistics (such as CHI², Fisher’s Exact Test)
	**4th step** Clinical signs at the day of decision making comparing the evaluation and empirical data set *(evaluation and empirical data set)*	àDescriptive and inferential statistics (such as CHI², Fisher’s Exact Test)
How do reasons for timely euthanasia differ regarding the evaluator’s profession?	Reasons reported by participants *(3rd survey question*,* single choice*,* evaluation data set)*	Descriptive and inferential statistics (such as CHI², Fisher’s Exact Test)
How do reasons for timely euthanasia differ regarding primary diagnosis?		

## Results

### Participants

The evaluators consisted of 30 practicing veterinarians (42.9%), 20 public official veterinarians (28.6%), and 17 farmers (21.5%). The remaining participants (*n* = 3; 4.7%) were affiliated with scientific or agricultural professions. Slightly more than half of the participants were female (52.9%), and the largest age group was 36–45 years (28.6%). Participants demonstrated substantial professional experience: 31.4% reported 16 or more years of experience working with pigs. Most participants had previous experience with euthanasia decisions, with 75.5% having decided to euthanize a compromised pig and 60.0% having conducted euthanasia themselves. In addition, 31.4% reported responsibility for training others in euthanasia procedures. 9.1% of participants were involved in the project before participating in the evaluation. Detailed demographic information is provided in Appendix D (“Demography”).

### Case groups

Based on responses to the first two evaluation questions, a total of 24 pigs were categorized as qualified euthanized pigs (QE), 72 pigs as qualified recovered pigs (QR) and 34 pigs as unclear cases (UC). Weaners represented about the majority of QE (54.3%) and QR group (47.2%), while weaner pigs (38.2%), fattening pigs (32.4%) and suckling pigs (29.4%) were more equally represented in the UC group.

In all groups, lameness or locomotor disorders were the common primary disorder (“PD”), (QE: 66.7%, QR: 34.7%, UC: 70.6%), while following PDs differed among groups (see Graphic 1). QE pigs were presented to evaluators an average of 2.5 times, with a maximum of eight examination day presentations. QR were presented to evaluators an average of 2.0 times (mean: 1.98), with a maximum of five presentations. UC were presented to evaluators an average of 2.4 times (mean: 2.37), with a maximum of seven presentations.

The presentation patterns of groups did not statistically differ (Kruskal–Wallis test: χ² = 0.00, *p* = 1.000). Furthermore, the categorization of pigs into QE, QR, or UC did not differ significantly between evaluator professions (χ² = 2.8875, *p* > .05), including the comparison of practicing veterinarians with public official veterinarians (χ² = 0.0602, *p* > .05).

**Fig. 1:**
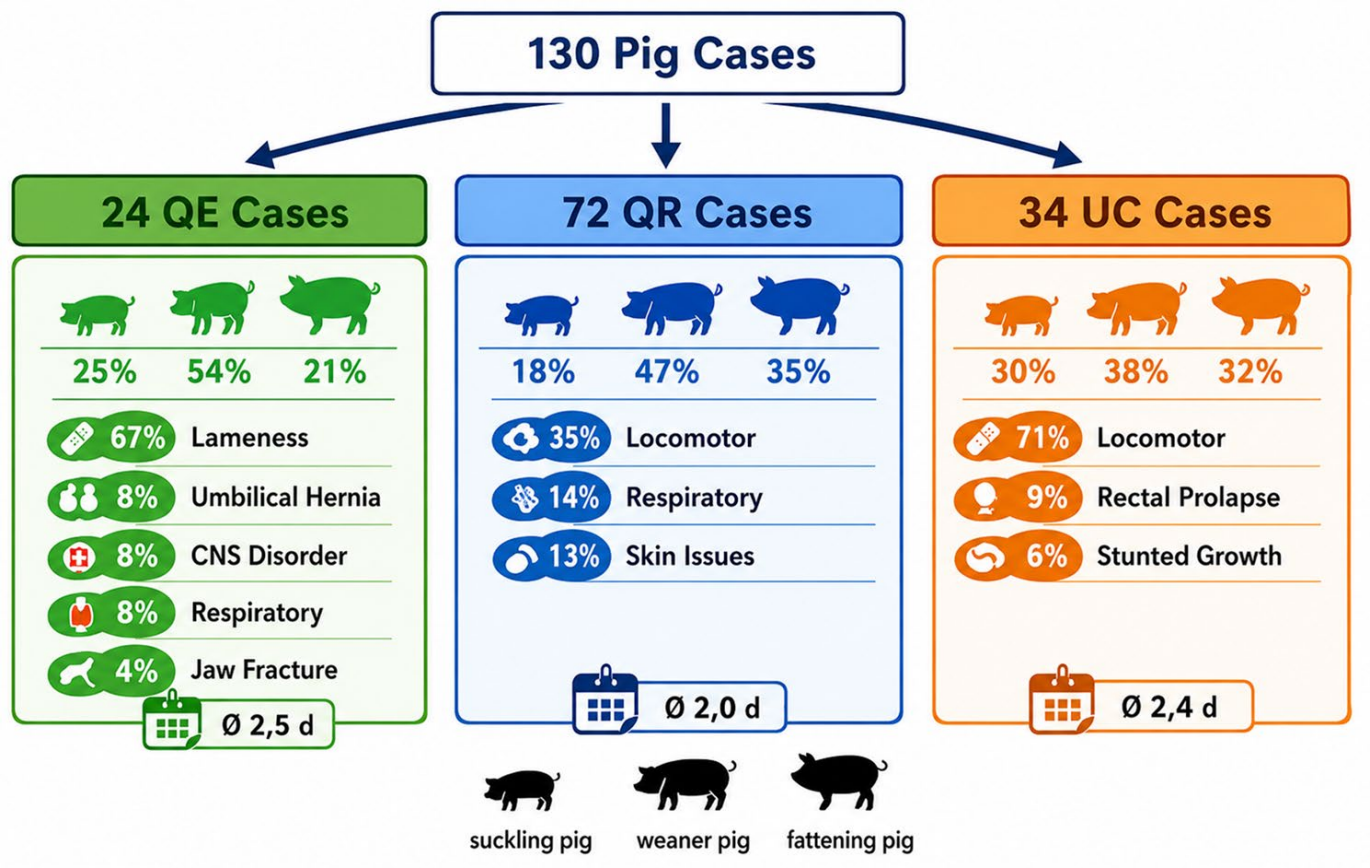
Overview of expert evaluation of pig cases

Comparing the decisions of evaluators and project veterinarians showed a mismatch of cases for four pigs actually euthanized in the project and nine recovered pigs. Not anticipating the discussion, the qualitative analysis suggests that the strict criteria used to define QE and QR categories make up the major part of discrepancies between evaluator classifications and empirical outcomes. Assessing decisions for UC pigs shows, for example, that for 24 of 34 UC cases, the general vote of evaluators (i.e. majority vote that did not exceed 65%) corresponded to the empirical decision made by the project veterinarians: 12 cases with a general vote in favor of euthanasia were actually euthanized in the project, and 12 cases with a general vote against euthanasia were actually recovered in the project). Comparing the decisions of evaluators and project veterinarians outlined that participants’ decisions differed from the empirical outcomes in 14.6% of cases, indicating a generally high level of correspondence between evaluator judgments and the decisions made in the project. Detailed results are provided in Appendix E (“Evaluation of Cases”).

### Reported reasons for euthanasia at the day of qualified euthanasia

Participants could indicate multiple reasons for their euthanasia decisions, including: one specific symptom, a combination of symptoms, severity of symptoms, overall health state, chance of treatment success, or duration of disease.

In 237 examination day presentations (16.3%), participants reported that **one specific symptom** guided their decision. Agreement was highest for broken mandibular bone (46.6%), and varied across primary diagnoses (PD) (χ² = 71.38, *p* < .0001). Shared clinical considerations across PDs (votes = 190) included: defining a specific diagnosis (26%), evaluating treatment effect (9%), non-ambulatory state (5%), difficulty accessing food (2%), and apathy (1%).

In 368 presentations (25.3%), **a combination of symptoms** guided decision making. Agreement ranged 23–27% for other PDs except broken mandibular bone, with significant differences between PDs (χ² = 15.77, *p* ≤ .01). Commonly cited aspects across PDs (514 votes) were low body condition (7%), poor overall health (6%), specific diagnosis (4%), and, in four PDs, retarded development (5%).

Consideration about the **severity of clinical signs** influenced euthanasia in 440 presentations (30.3%). Highest agreement (~ 31%) was observed for locomotor, CNS, and respiratory disorders, with no significant differences between PDs (*p* > .05). Shared considerations (496 votes) included low body condition (6%), specific diagnosis (6%), treatment effect (7%), and poor overall health (4%).

In 355 presentations (24.4%), decisions were based on the pig’s **overall health status**. Agreement was highest for respiratory disorders, with significant variation across PDs (χ² = 15.85, *p* < .01). Shared aspects (369 votes) included specific diagnosis (26%), treatment effect (9%), non-ambulatory state (5%), difficulty accessing food (2%), and apathy (1%).

Evaluators cited the role of **treatment success** in 594 cases (40.8%). Highest agreement occurred for broken mandibular bone (46.6%) and locomotor disorders (43.4%), with significant variation across PDs (χ² = 23.88, *p* < .0001). Follow-up responses indicated a grave prognosis in over 75% of cases across all PDs.

Considerations about **the duration of disease** guided decisions in 339 presentations (23.3%), particularly for respiratory (39.7%) and CNS disorders (37%), with significant differences among PDs (χ² = 60.61, *p* < .0001). Participants most frequently indicated that euthanasia would be timely on the **second presentation day** (36.3%), with others suggesting waiting 2–3 days (57%) or 4–7 days (16.9%). Only 3.1% judged the signs uncritical for a decision to euthanize the pig. Notably, at the day of qualified timely euthanasia (QE), duration was not cited as a reason, and no follow-up question on waiting period was asked.

Across QE pigs, the **chance of treatment success** was the most frequently cited reason (440 presentations). Free-text responses further highlighted the clinical signs of low body condition, poor overall health, and specific diagnosis as common considerations across PDs. Comparing responses across professions, veterinarians cited **chance of treatment success** significantly more often than farmers (*n* = 1456; Fisher’s exact test, *p* < .001), though this difference did not vary by primary diagnosis. Similarly, **severity of clinical signs** was reported more frequently by veterinarians (*n* = 1456; χ² = 23.57, *p* < .0001). All reported clinical aspects and an illustration of the most frequently shared ones are provided in Appendix F (“Evaluation of cases”).

When comparing **practicing versus public official veterinarians**, a significant difference was observed only for the reason **one specific symptom** (Fisher’s exact test, *p* = .0001), while no significant differences were found for combinations of clinical signs. Overall, the decision whether and when to euthanize did not differ significantly between any of these groups. See Tables and illustrating Figs. 1, 2, 3, 4 and 5 in Appendix F and G (“Comparing reasons for euthanasia”).

### Reported reasons for euthanasia at the day of qualified euthanasia

To complement the evaluators’ reported reasons for classifying cases as QE, QR, or UC, the empirical dataset was analysed with particular focus on the clinical signs identified in the previous sections. The analysis concentrated on clinical signs most frequently reported across the five primary diagnoses CNS disorders, locomotor disorders, umbilical outpouching, respiratory disorders, and bone fractures. Initial interpretive considerations are presented in this section to facilitate the subsequent discussion.

A **poor overall health state**, one of the most frequently reported reasons for euthanasia, differed significantly between case groups in the empirical dataset (χ² = 41.2, *p* < .0001). This condition was recorded at least once in more than 65% presentations of both QE and UC cases, suggesting that the timing and persistence, rather than the mere presence of this sign, may be critical for euthanasia decisions. The overall health assessment often represents a summary of the clinical examination. This includes parameters such as rectal temperature, which also differed significantly between case groups (χ² = 8.6, *p* < .05). Additionally, long bristles, indicating a prolonged disease course, showed group differences (χ² = 8.01, *p* < .05), which may be used to increase objectivity when referring to the poor overall health status of a pig in the future.

Another commonly shared sign across PDs was low body condition, which differed significantly between case groups (χ² = 22.4, *p* < .0001). Similarly, retarded development (χ² = 13.5, *p* < .01) and abnormal behaviour (including apathy; χ² = 13.4, *p* < .01) were associated with group differences. Difficulty accessing feed showed a marginal association (χ² = 5.95, *p* = .05). In contrast, hunched back, rarely cited by evaluators as a reason for euthanasia, was most prevalent in UC cases (8.2% overall; 28.4% of UC cases; χ² = 15.25, *p* < .001).

Given the large proportion of locomotor disorders in the dataset, additional analyses focused on lameness-related clinical signs. While evaluators frequently referred to “lameness,” this term is unspecific and equals the recorded sign **abnormal gait** in the dataset. The analysis emphasizes that groups did not differ significantly in this regard (χ² = 2.6, *p* > .05). More specific clinical signs provided better discrimination, including relief of limb(s) (χ² = 17.08, *p* < .001), affected locomotion phase (χ² = 21.93, *p* < .001), and hindlimb paresis (χ² = 15.47, *p* < .001). Interestingly, claw lesions were more common in QR pigs (11.3% overall; 27.9% within QR; χ² < 0.0001), while the term rather describes an aetiology of lameness.

A non-ambulatory state also differed between case groups (χ² = 8.16, *p* < .05). Earlier indicators, rarely mentioned by evaluators but recorded in the empirical data, included carpal weight bearing or knuckling (χ² = 7.76, *p* < .05) and difficulty rising (χ² = 15.18, *p* < .05).

Disease-specific signs were also assessed. Significant differences were observed for tail-biting lesions (χ² = 10.83, *p* < .01) and lesion severity (χ² = 29.29, *p* < .0001). In contrast, size of umbilical outpouching did not differ significantly (χ² = 5.08, *p* > .05), although large ventrally extending umbilical outpouchings occurred only in euthanized pigs. No significant group differences were found for dyspnea (χ² = 0.23, *p* > .05) or enlarged joints (χ² = 1.78, *p* > .05). Detailed results are provided in Appendix H.

### Comparison of evaluation and empirical dataset

To determine whether clinical signs identified by evaluators corresponded with those observed on the empirical day of euthanasia, the 24 QE cases were further analysed. Four cases (one fracture and three locomotor disorders) that evaluators considered for euthanasia but that later recovered in the project were excluded.

Across all remaining variables, no significant differences were found between the clinical signs recorded on the day of qualified euthanasia and those on the empirical euthanasia day. A second analysis including all examination days with ≥ 50% agreement for euthanasia also revealed no significant differences. These findings indicate that clinical signs identified in the evaluation dataset correspond well with those observed in the empirical dataset, supporting the transferability of the derived euthanasia criteria (Appendices I and J).

### Synthesis and discussion

The results indicate that timely euthanasia decisions depend on the primary diagnosis but share several common considerations including clinical signs. Based on evaluator responses and empirical clinical data, four key criteria can be identified – a thorough examination of clinical signs (general health, locomotion, disease-specific signs), establishing a specific diagnosis, considering the chance of treatment success and defining an appropriate decision timeframe in this regard.

#### Clinical examination and relevant clinical signs

Timely euthanasia decisions require systematic clinical examination of individual pigs. A poor overall health state and abnormal behavior is frequently reported and recorded for timely euthanized pigs, but should be further specified with the help of parameters such as rectal temperature, body condition, development state, and apathy from the first detection of a compromised health state. Locomotion assessment is particularly important in cases with locomotor disorders. While reduced mobility or inability to stand is often summarised as “severe lameness” or “non-ambulatory state”, more specific descriptions, such as the amount of relieved limbs, difficulty rising, or affected phases of lameness provide clearer clinical information. Notably, a non-ambulatory state does not automatically necessitate euthanasia on day one, as indicated by majority votes, provided adequate care and treatment are possible. Disease-specific signs (e.g., size of umbilical outpouchings, joint swelling, or respiratory distress) were less frequently decisive alone but help monitor disease progression and treatment response.

#### Specific diagnoses

The second criterion is the **definition of a specific diagnosis**. In practice, diagnoses were often based on primary clinical signs (e.g., pumping respiration or lameness). However, more precise biomedical diagnoses, such as fractures, septic arthritis, or bacterial pneumonia, allow a clearer prognosis and better treatment planning. Improved diagnostic specificity may also encourage additional diagnostic procedures in uncertain cases, reducing delays and uncertainty in euthanasia decisions [11, 14].

#### Chance of treatment success

In view of the specific diagnosis, the **chance of treatment success** is central to determining the appropriate time for euthanasia. This requires that compromised pigs receive an initial treatment attempt and that treatments are documented for follow-up evaluations. In the project, treatment plans including early and continuous analgesia were associated with improved recovery (see [9, 10]). In this study, farmers considered treatment success less frequently than veterinarians, suggesting that veterinarians should communicate their reasoning more explicitly to share experiences. Documentation of treatments should also be harmonized between farmers and veterinarians in accordance with EU Regulation 2019/6 (Art. 105(5) [4] and national veterinary regulations (e.g. TÄHV, [16]). Nevertheless, euthanasia remains a **case-by-case decision**. When a diagnosis clearly indicates a poor prognosis, such as fractures of weight bearing bones, immediate euthanasia may be appropriate without prior treatment.

#### Decision timeframe

A final key factor is the timeframe for re-assessing compromised pigs. In the evaluation dataset, most votes for timely euthanasia occurred at the second presentation, with suggestions that euthanasia should have occurred earlier often appearing by the second or third presentation. In the project, pigs were typically re-examined every 2–3 days. Taken together, the results suggest that euthanasia decisions are commonly reached within two to three re-evaluations (approximately 2–6 days) after initial identification. This timeframe allows treatment attempts while avoiding prolonged suffering. In the future, the potential of telemedicine could be explored to monitor the progression of clinical signs within this timeframe and to overcome the structural and biosafety challenges associated with veterinarians accessing different herds over time.

Overall, the findings of this analysis align with earlier studies recommending **re-evaluation within 24–48 h** once critical clinical signs are detected [13]. They also support the consensus that euthanasia decisions should be based on **clusters of clinical signs rather than single indicators**, including general health status, locomotion, access to feed and water, and wound characteristics [13, 15]. Unlike previous studies relying mainly on expert opinion, this study integrates **expert evaluation with empirical on-farm clinical data**, providing stronger evidence for practical euthanasia criteria [2, 3, 7, 17].

The limitations of the study include that results primarily represent the perspectives of experienced German pig professionals, given that participants were primarily veterinarians, with fewer farmers and few individuals under 25 years of age, despite that response patterns were largely consistent across professions.

The empirical dataset originated from five conventional pig farms, with a predominance of locomotor disorders. While previous studies emphasize the high prevalence of lameness for sows in particular [8], our study highlights the high prevalence for other age categories. While for our results, this high prevalence may influence the distribution of clinical signs, the proposed euthanasia criteria extend beyond locomotor disorders and are likely applicable to other pig categories and production systems.

Finally, evaluators assessed 376 case presentations representing 130 pigs, resulting in more than 22,000 individual evaluations. Although evaluator decisions corresponded well with empirical outcomes, case presentations represented only selected examination days, and the overall proportion of assessed cases was limited. Further analyses and validation studies are therefore necessary before broad generalization.

## Conclusions

To safeguard the welfare of compromised pigs, veterinarians and farmers must ensure timely euthanasia decision-making. Based on more than 22,000 evaluated pig examination-day presentations, this study defines criteria for timely euthanasia decisions.

First, veterinarians and farmers must perform a thorough clinical examination and repeated re-examinations within a timeframe of approximately 2–6 days. The occurrence and progression of clinical signs should be systematically recorded and documented. Using unspecific terms such as lameness or non-ambulatory state alone are insufficient for the early identification of pigs that may require euthanasia. Instead, more specific antecedent clinical signs, such as difficulty rising, or specific phases of impaired locomotion, should be carefully observed and monitored over time.

Second, a specific diagnosis should be established. This should ideally include consideration of the underlying patho-aetiological process, for example lameness caused by claw lesions or bacterial pneumonia leading to dyspnea. Such diagnostic specification is essential because it informs prognosis and clinical management.

Finally, the chance of treatment success must be evaluated in relation to the specific diagnosis. The decision whether to continue treatment or proceed with euthanasia should therefore be based on both the expected prognosis and the documented development of clinical signs during the observation period.

Overall, this study highlights that the terminology used to describe clinical signs and the general health status of pigs requires greater standardization. This point could be addressed through training programs for veterinarians and practitioners involved in pig herd health management. Improved and consistent terminology would also facilitate more robust scientific analyses and support clearer clinical reasoning and decision-making in farm practice.

## Supplementary Information

Below is the link to the electronic supplementary material.

Supplementary Material 1

## Data Availability

The data obtained were processed and evaluated anonymously and in compliance with the EU’s General Data Protection Regulation as well as with all data privacy regulations from Germany and the Federal State of Lower Saxony. The data are not publicly available due to ethical and privacy restrictions, i.e., any data transfer to interested persons is not allowed without a specific formal contract. This contract is available to qualified researchers and will include guarantees of the obligation to maintain data confidentiality in accordance with the provisions of the German data protection law. Currently, there is no data access committee or another body that could be contacted for the data. However, for this purpose, a committee will be founded. This future committee will consist of the authors as well as members of the University of Veterinary Medicine Hannover and members of the funding institution. Interested cooperative partners who are able to sign a contract as described above may contact the corresponding author.
