# Supplementary material for: Criteria for timely euthanasia of compromised pigs based on expert opinion and longitudinal data collection on farm
Source: Porcine Health Manag. 2026 Aug 8;12:44. doi: 10.1186/s40813-026-00532-2 (PMC13455467; doi:10.1186/s40813-026-00532-2)
Supplement: Supplementary file 1 [file 40813_2026_532_MOESM1_ESM.docx]

## Appendix

### A Original data set

##### Table A1: Class of pigs in the project

| **Class** | **Frequency** | **Percentage (%)** |
| --- | --- | --- |
| **Suckling piglets** | 758 | 23.17 |
| **Weaner** | 1405 | 42.94 |
| **Fattening pig** | 1086 | 33.19 |
| **Sows** | 23 | 0.70 |
| **Sum** | 3272 | 100% |

##### Table A2: Primary diagnoses of pigs

| **Primary Diagnoses (I/II)** | **Frequency** | **%** |  | **Primary Diagnoses (II/II)** | **Frequency** | **%** |
| --- | --- | --- | --- | --- | --- | --- |
| **Loss of weight** | 18 | 0.55 |  | **Tail lesion** | 289 | 8.83 |
| **Diarrhea** | 19 | 0.58 |  | **CNS disorder** | 120 | 3.67 |
| **Retarded development** | 66 | 2.02 |  | **Other** | 2 | 0.06 |
| **Non-ambulatory (due to lameness)** | 12 | 0.37 |  | **Abscesses (increased circumference)** | 12 | 0.37 |
| **Non-ambulatory (due to CNS disorder)** | 11 | 0.34 |  | **Respiratory / Circulatory system disorder** | 14 | 0.43 |
| **Skin disorder** | 158 | 4.83 |  | **Ocular disorder** | 17 | 0.52 |
| **Skin injury** | 136 | 4.16 |  | **Locomotory tract disorder** | 15 | 0.46 |
| **Hindlimb paresis / paralysis** | 28 | 0.86 |  | **Bad overall health status** | 21 | 0.64 |
| **Broken bone** | 13 | 0.40 |  | **Infections** | 1 | 0.03 |
| **Lameness** | 1985 | 60.67 |  | **Broken mandibular jaw** | 17 | 0.52 |
| **Pneumonia** | 62 | 1.89 |  | **Outpouching  (other than umbilical)** | 5 | 0.15 |
| **Rectal prolapse** | 57 | 1.74 |  | **Pumping breath** | 83 | 2.54 |
| **Umbilical outpouching** | 111 | 3.39 |  |  |  |  |

##### Table A3: Average visitations and duration of pigs in the project

|  | **Variable** | **Mean** | **SD** | **Minimum** | **Maximum** |
| --- | --- | --- | --- | --- | --- |
| **All pigs** | **Time in the project**; **Visitation** | 10.9; 4.2 | 12.02; 3.2 | 1.0; 1.0 | 113.0; 23.0 |
| **Euthanized pigs** | **Time in the project**; **Visitation** | 11.5; 4.2 | 13.34; 3.07 | 1.0; 1.0 | 99.0; 16.0 |

##### Table A4: Categorization of pigs in the project

|  | **Frequency** | **%** |
| --- | --- | --- |
| **Euthanized** | 80 | 13.01 |
| **Recovered / not euthanized** | 535 | 86.99 |
| **Sum** | 615 | 100 |

##### Table A5: Categories of pigs per age

|  | **Suckling pigs** | **Weaners** | **Fattening pigs** | **Sow** | **total** |
| --- | --- | --- | --- | --- | --- |
| **Euthanized** | 92 | 186 | 142 | 0 | 420 (12.86%) |
| **Recovered / not euthanized** | 666 | 1219 | 944 | 23 | 2852 (86.99%) |
| **Sum** | 758 | 1405 | 1086 | 23 | 3272 |

### B Evaluation data set

##### Table B1: Categories of pigs per primary diagnoses

| **Primary diagnosis** | **Suckling pig** | **Weaner** | **Fattening pig** | **Total (%)** |
| --- | --- | --- | --- | --- |
| **Loss of weight** | 0 | 0 | 3 | 3 (0.8) |
| **Diarrhea** | 0 | 1 | 3 | 4 (1.1) |
| **Retarded development** | 2 | 9 | 6 | 17 (4.5) |
| **Non-ambulatory (due to lameness)** | 0 | 0 | 1 | 1 (0.3) |
| **Skin disorder** | 3 | 12 | 10 | 25 (6.65) |
| **Integument injury** | 5 | 3 | 2 | 10 (2.7) |
| **Broken bone** | 1 | 1 |  | 2 (0.4) |
| **Rectal prolapse** | 0 | 3 | 4 | 7 (1.9) |
| **Umbilical outpouching** | 0 | 9 | 3 | 12 (3.2) |
| **Tail lesion** | 1 | 9 | 18 | 28 (7.5) |
| **CNS disorder** | 0 | 25 | 1 | 26 (6.9) |
| **Other** | 2 | 9 | 4 | 15 (3.99) |
| **Lameness / Locomotor tract** | 67 | 70 | 56 | 193 (51.3) |
| **Respiratory system**  **(pneumonia, pumping breath)** | 3 | 19 | 11 | 33 (8,9) |
| **Sum of clinical examinations** | 84 | 170 | 122 | 376 |

##### Table B3: Average visitations and duration of pigs in the evaluation

| **Pig category** | **Variable** | **Mean** | **SD** | **Minimum** | **Maximum** |
| --- | --- | --- | --- | --- | --- |
| **All pigs** | **Time in the project**; **Visitation** | 7.6; 3.1 | 11.02; 2.69 | 1; 1.0 | 69.0; 18 |
| **Euthanized pigs** | **Time in the project**; **Visitation** | 6.5; 2.8 | 8.3; 2.1 | 1.0; 1.0 | 40.0; 16.0 |

##### Table B4: Categories of euthanized pigs per primary diagnoses (n=absolute frequency)

| **Primary diagnosis** | **Suckling pig** | **Weaner** | **Fattening pig** | **Total (%)** |
| --- | --- | --- | --- | --- |
| **Loss of weight** | 0 | 0 | 2 | 2 (1.3) |
| **Retarded development** | 0 | 0 | 5 | 5 (3.3) |
| **Non-ambulatory (due to lameness)** | 0 | 0 | 1 | 1 (0.7) |
| **Skin disorder** | 0 | 0 | 3 | 3 (1.97) |
| **Integument injury** | 0 | 0 | 1 | 1 (0.7) |
| **Umbilical outpouching** | 0 | 9 | 0 | 9 (5.9) |
| **Tail lesion** | 0 | 6 | 9 | 15 (9.9) |
| **CNS disorder** | 0 | 5 | 0 | 5 (3.3) |
| **Other** | 0 | 0 | 2 | 2 (1.3) |
| **Lameness / Locomotor tract** | 37 | 47 | 15 | 99 (65.1) |
| **Respiratory system**  **(pneumonia, pumping breath)** | 0 | 6 | 4 | 10 (6.6) |
| **Sum** | 37 | 73 | 42 | 152 |

##### Table B5: Median presented amount of visitations

|  | **N** | **Sum of Scores** | **Expected Under H0** | **Std Dev Under H0** | **Mean Score** |
| --- | --- | --- | --- | --- | --- |
| **QR** | 72 | 4716.0 | 4716.0 | 0.0 | 65.50 |
| **QE** | 24 | 1572.0 | 1572.0 | 0.0 | 65.50 |
| **UC** | 34 | 2227.0 | 2227.0 | 0.0 | 65.50 |

**Statistic for table B5: Kruskal-Wallis Test: 0.000, p-value: 1.000.**

### C Conduct of evaluation

##### C1 Original case presentations

Graphic 1: Graphical Case Presentation

##### C2 Overview of evaluation questions in English language

1.1. **What decision do you make for this pig on base of the provided information [obligatory, single choice]**
- No (further) action
- intensified observation without separation
- Intensified observation and additional separation
- Treatment without separation
- Treatment and additional separation
- Euthanasia
- Other answer

*1.2. In case of 1.1 == Euthanasia*

- **How do you evaluate the time point of euthanasia [obligatory, single choice]**
  - Timely
  - Too late
  - Other answer
  2.1. **Why do you come to this decision? [obligatory, multiple choice]**
       - A specific symptom
       - Combination of specific symptoms
       - Severity of specific symptoms
       - Overall condition of the pig
       - Consideration of treatment success
       - Duration the pig has been ill
       - Other answer

*2.2.1 In case of 2.1. == a specific symptom*

- **What specific symptom is decisive? [obligatory, free text]**

*2.2.2 In case of 2.1. == combination of specific symptoms*

- **What combination of symptoms is decisive? [obligatory, free text]**

*2.2.3 In case of 2.1. == severity of specific symptoms*

- **Which symptoms best indicate the severity to you? [obligatory, free text]**

*2.2.4 In case of 2.1. == overall condition of the pig*

- **Please describe the “overall health status” in own words [obligatory, free text]**

*2.2.5 In case of 2.1. == Consideration of treatment success*

- **How do you assess the success of (continuing to) treat the pig at this point?**
  (additional information: In this case, you would have the freedom to choose the treatment/medication yourself (taking into account previous treatment instructions).) [obligatory, single choice]
  - Good
  - Moderate
  - Grave
  - Other answer

2.2.6 In case of 2.1. == Duration the pig has been ill

- **How long can the pig continue to exhibit the described symptoms without making your decision more critical? [obligatory, single choice]**
  - Not another day - simply finding the pig with the symptoms is critical.
  - 1 day
  - 2 days
  - 3 days
  - 4-7 days
  - 8-14 days
  - 15-21 days
  - Until further notice - the presence of symptoms is not critical.
  - Other answer

**2.7 Which materials helps you making a decision [optional]**

- Written information
- Pictures, footage
- Videos
- No answer

**2.8 In these fields you may make own notes [optional, free text]**

- Notes about the case
- Notes to questions or answers
- other

### D Demography

##### Table D1: Profession of participants

| Profession | Position | n | % |
| --- | --- | --- | --- |
| Farmer | Agricultural setting | 6 | 8.6 |
|  | Farmer | 9 | 12.9 |
|  | Animal husbandry | 2 | 1.4 |
| Veterinarians | Scientific / institutional background |  | 4.3 |
|  | Public official veterinarian | 20 | 28.6 |
|  | Tierarzt / Tierärztin (praktizierend) | 30 | 42.9 |
| Total | | 70 | 100.0 |

##### Table D2: Age of participants

| Age | n | percent |
| --- | --- | --- |
| . | 14 | 20.00 |
| 18-25 y | 1 | 1.43 |
| 26-35 y | 15 | 21.43 |
| 36-45 y | 20 | 28.57 |
| 46-55 y | 12 | 17.14 |
| >= 56 y | 8 | 11.43 |
| Total | 70 | 100.00 |

##### Tabel D3: Gender of participants

| Gender | n | percent |
| --- | --- | --- |
| Male | 33 | 47.14 |
| Female | 37 | 52.86 |
| Total | 70 | 100.00 |

##### Table D4: Years of experiences of participants

| Experience with pigs | n | percent |
| --- | --- | --- |
| . | 16 | 22.86 |
| 1-5 y | 8 | 11.43 |
| 6-10 y | 18 | 25.71 |
| 11-15 y | 6 | 8.57 |
| 16 or more years | 22 | 31.43 |
| Total | 70 | 100.00 |

##### Table D5: Experience and roles of participants

|  | n | % |
| --- | --- | --- |
| Responsibility of making the decision to euthanize |  |  |
| . | 13 | 18.6 |
| Yes | 46 | 65.7 |
| No | 11 | 15.7 |
| Have already decided to euthanize a pig |  |  |
| . | 13 | 18.6 |
| Yes | 53 | 75.7 |
| No | 4 | 5.7 |
| Respondibility of conducting euthanasia |  |  |
| . | 13 | 18.6 |
| Yes | 28 | 40.0 |
| No | 29 | 41.4 |
| Having performed euthanasia of a pig |  |  |
| . | 13 | 18.6 |
| Yes | 42 | 60.0 |
| No | 15 | 21.4 |
| Responsibility to control euthanasia of pigs |  |  |
| . | 13 | 18.6 |
| Yes | 29 | 41.4 |
| No | 28 | 40.0 |
| Responsibility to control euthanasia decision / conduct of others |  |  |
| . | 13 | 18.6 |
| Yes | 22 | 31.4 |
| No | 35 | 50.0 |

##### Table D6: Participation in euthanasia related projects or activities

|  | n | % |
| --- | --- | --- |
| Previous activities of the project CARE-PIG |  |  |
| missing | 13 | 18.6 |
| Yes | 6 | 8.6 |
| No | 51 | 72.9 |
| Participating in data collection |  |  |
| Missing / question not posed (only if involved in the project) | 64 | 91.5 |
| Yes | 2 | 2.9 |
| No | 4 | 5.7 |
| An Umfrage teilgenommen |  |  |
| Missing / question not posed (only if involved in the project) | 64 | 91.5 |
| Yes | 1 | 1.4 |
| No | 5 | 7.1 |
| Participation in previous projects |  |  |
| Missing / question not posed (only if involved in the project) | 64 | 91.5 |
| Yes | 3 | 4.3 |
| No | 3 | 4.3 |
| Other project activities |  |  |
| Missing / question not posed (only if involved in the project) | 64 | 91.5 |
| Yes | 1 | 1.4 |
| No | 5 | 7.1 |

##### Table D7: Evaluated case presentation by profession

|  | Number of evaluated case presentations | % |
| --- | --- | --- |
| Farmer | 3414 | 15.0 |
| Veterinarians | 19310 | 85.0 |
| Total | 22724 | 100.0 |

### E Evaluation of cases

##### Table E1: Comparison of evaluators’ and project veterinarians’ decisions

| **Decision in the project**  n  row pct  col pct | **Qualified euthanized (QE)** | **Qualified recovered (QR)** | **Unclear cases**  **(UC)** | **Sum** |
| --- | --- | --- | --- | --- |
| **Euthanized** | 20 | 9 | 22 | 51 |
|  | 0.83 | 0.13 | 0.92 |  |
|  | 0.39 | 0.18 | 0.43 |  |
| **Recovered /**  **not euthanized** | 4 | 63 | 12 | 79 |
|  | 0.17 | 0.88 | 0.35 |  |
|  | 0.05 | 0.8 | 0.15 |  |
| **SUM *n*** | 24 | 72 | 34 | 130 |

##### Table E2: Votes of timely euthanasia (n=absolute frequency)

| **Decision** | **Number of Visitation** | | | | | | | | **SUM** |
| --- | --- | --- | --- | --- | --- | --- | --- | --- | --- |
|  | **1** | **2** | **3** | **4** | **5** | **6** | **7** | **8** |  |
| **Timely** | 729 | 1002 | 645 | 238 | 68 | 17 | 49 | 16 | 2764 (56.6) |
| **Consider earlier** | 242 | 567 | 555 | 432 | 161 | 49 | 39 | 36 | 2081 (42.62) |
| **Other answer** | 8 | 8 | 12 | 4 | 1 | 2 | 2 | 1 | 38  (0.8) |
|  |  |  |  |  |  |  |  |  |  |
| **Total** | 979 | 1577 | 1212 | 647 | 230 | 68 | 90 | 53 | 4883 |

##### Table E3: Comparison of evaluators’ categorization by profession

| **Profession**  n | **Qualified euthanized (QE)** | **Qualified recovered (QR)** | **Unclear cases**  **(UC)** | **Sum** |
| --- | --- | --- | --- | --- |
| Farmer | 747 | 1722 | 945 | 3414 |
| Veterinarian | 4058 | 9653 | 5599 | 19310 |
|  |  |  |  |  |
| **Total** | 4805 | 11375 | 6544 | 22724 |

**Statistic for table E3: CHI²-value 2.8875, p-value: 0.2360.**

##### Table E4: Comparison of veterinarians’ categorization by background

| **Profession**  n | **Qualified euthanized (QE)** | **Qualified recovered (QR)** | **Unclear cases**  **(UC)** | **Total** |
| --- | --- | --- | --- | --- |
| Public official veterinarian | 1495 | 3577 | 2070 | 7142 |
| Practicing veterinarian | 2326 | 5512 | 3202 | 11040 |
|  |  |  |  |  |
| **Total** | 3821 | 9089 | 5272 | 18182 |

**Statistic for table E4: CHI²-value: 0.0602, p-value: 0.9703.**

##### Table E5: Comparison of primary diagnosis for QE pigs

| **Primary diagnosis** | **Suckling pig** | **Weaner** | **Fattening pig** | **Total (%)** |
| --- | --- | --- | --- | --- |
| Broken bone | 1 | 0 | 0 | 1 (4.17) |
| Umbilical outpouching | 0 | 2 | 0 | 2 (8.33) |
| Tail lesion | 0 | 1 | 0 | 1 (4.17) |
| CNS disorder | 0 | 2 | 0 | 2 (8.3) |
| Locomotor tract | 5 | 7 | 4 | 16 (66.67) |
| Respiratory system  (pneumonia, pumping breath) | 0 | 1 | 1 | 2 (8.33) |
|  |  |  |  |  |
| **Total** | 6 | 13 | 5 | 24 |

##### Table E6: Comparison of primary diagnosis for QR pigs

| **Primary diagnosis** | **Suckling pig** | **Weaner** | **Fattening pig** | **Total (%)** |
| --- | --- | --- | --- | --- |
| Loss of weight | 0 | 0 | 1 | 1 (1.39) |
| Diarrhea | 0 | 1 | 2 | 3 (4.17) |
| Retarded development | 1 | 1 | 2 | 2 (2.78) |
| Non-ambulatory (due to lameness) | 0 | 0 | 1 | 1 (1.39) |
| Skin disorder | 1 | 5 | 3 | 9 (12.5) |
| Integument injury | 2 | 1 | 0 | 3 (4.17) |
| Broken bone | 0 | 1 | 0 | 1 (1.39) |
| Umbilical outpouching | 0 | 1 | 1 | 2 (2.78) |
| Tail lesion | 0 | 1 | 2 | 3 (4.17) |
| CNS disorder | 0 | 6 | 0 | 6 (8.33) |
| Other | 1 | 3 | 2 | 6 (8.33) |
| Lameness / Locomotor tract | 7 | 9 | 9 | 25 (34.72) |
| Respiratory system  (pneumonia, pumping breath) | 1 | 5 | 4 | 10 (13.89) |
|  |  |  |  |  |
| **Total** | 13 | 34 | 25 | 72 |

##### Table E7: Comparison of primary diagnosis for UC pigs

| **Primary diagnosis** | **Suckling pig** | **Weaner** | **Fattening pig** | **Total (%)** |
| --- | --- | --- | --- | --- |
| Loss of weight | 0 | 0 | 1 | 1 (2.94) |
| Retarded development | 0 | 1 | 1 | 2 (5.88) |
| Skin disorder | 0 | 0 | 1 | 1 (2.94) |
| Rectal prolapse | 0 | 1 | 2 | 3 (8.82) |
| Tail lesion | 0 | 0 | 1 | 1 (2.94) |
| CNS disorder | 0 | 1 | 0 | 1 (2.94) |
| Lameness / Locomotor tract | 10 | 9 | 5 | 24 (70.59) |
| Respiratory system  (pneumonia, pumping breath) | 0 | 1 | 0 | 1 (2.94) |
|  |  |  |  |  |
| **Total** | 10 | 13 | 11 | 34 (100) |

##### Table E8: Mean visitation of case groups

| **Pig category** | **Mean** | **SD** | **Minimum** | **Maximum** |
| --- | --- | --- | --- | --- |
| QE | 2.5 | 1.58 | 1.0 | 8 |
| QR | 1.9 | 0.97 | 1.0 | 5 |
| UC | 2.4 | 1.38 | 1 | 7 |

**Statistics for Table E8: Chi²-value: 23.72, p-value 0.0495.**

##### Table E9: Comparison of evaluated cases per profession

|  | **QE** | **QR** | **UC** | **Total** |
| --- | --- | --- | --- | --- |
| Veterinarian | 4058 | 9,653 | 5,599 | 19,310 |
| Farmer | 747 | 1722 | 945 | 3,414 |
| **Total clinical examinations** | 4,805 | 11,375 | 6,544 | 22,724 |

**Statistics for Table E9: CHI²-value: 2.8875, p-value: 0.236.**

##### Table E10: Comparison of groups per profession veterinarians

|  | **QE** | **QR** | **UC** | **Total** |
| --- | --- | --- | --- | --- |
| Veterinarian (public official) | 1495 | 3577 | 2070 | 7142 |
| Veterinarian (practicing) | 2326 | 5512 | 3202 | 11040 |
| **Total clinical examinations** | 3821 | 9089 | 5272 | 18182 |

**Statistics for Table E9: CHI²-value: 0.602, p-value: 0.9703.**

##### Table E11: Comparison of decisions from evaluators and project veterinarians

| **Empirical case group………….**  n  row pct  col pct | **Evaluated case groups** | | | |
| --- | --- | --- | --- | --- |
|  | **Qualified euthanized**  **(QE)** | **Qualified recovered (QR)** | **Unclear**  **cases**  **(UC)** | **Total** |
| **euthanized** | 20 | 9 | 22 | 51 |
|  | 0.39 | 0.18 | 0.43 | 0.95 |
|  | 0.83 | 0.13 | 0.92 |  |
| **recovered / not euthanized** | 4 | 63 | 12 | 79 |
|  | 0.05 | 0.8 | 0.15 | 1.0 |
|  | 0.17 | 0.88 | 0.35 |  |
|  | 24 | 72 | 34 | 130 |
| (iterated cases n=3 excluded since similar decisions (recovery) were made) | | | | |

### F Comparing reasons for euthanasia

#### F1 Specific clinical aspects

##### Table F1.1: Comparing the vote for “specific clinical aspects” per primary diagnosis

| Frequency  Percent  Row pct  Col pct | **Primary diagnosis** | | | | | |
| --- | --- | --- | --- | --- | --- | --- |
|  | **Broken Bones** | **Umbilical Outpouching** | **CNS disorder** | **Locomotor disorder** | **Respiratory tract disorder** | **Total** |
| **Yes** | 27; 1.85; 11.39; 46.55 | 39; 2.68; 16.46; 32.23 | 13; 0.89; 5.49; 10.92 | 146; 10.03; 61.60; 14.08 | 12; 0.82; 5.06; 9.92 | 237; 16.28 |
| **No** | 31; 2.13; 2.54; 53.45 | 82; 5.63; 6.73; 67.77 | 106; 7.28; 8.70; 89.08 | 891; 61.20; 73.09; 85.92 | 109; 7.49; 8.94; 90.08 | 1219; 83.72 |
| **Total** | 58; 3.98 | 121; 8.31 | 119; 8.17 | 1037; 71.22 | 121; 8.31 | 1456; 100.00 |

**Statistics for Table F1: CHI²-value: 71.3778, p-value: <.0001**

##### Table F1.2: Summary of open reported “specific clinical aspects” for PD: CNS disorders

|  | **n** | **%** |
| --- | --- | --- |
| Non-ambulatory | 4 | 0.44 |
| Severity state | **2** | 0.22 |
| Treatment effect | 2 | 0.22 |
| Diagnosis CNS | 1 | 0.11 |
|  |  |  |
| **Total** | 9 | 0.99 |

##### Table F1.3: Summary of open reported “specific clinical aspects” for PD: locomotor tract

|  | **n** | **%** |
| --- | --- | --- |
| Additional Diagnoses | 3 | 0.03 |
| Diagnosis | 19 | 0.16 |
| Hindlimb-Paralysis/Paresis | 13 | 0.11 |
| Hunched back | 1 | 0.01 |
| Infection | 3 | 0.03 |
| Joint hard. red | 2 | 0.02 |
| Lameness | 31 | 0.26 |
| No food | 1 | 0.01 |
| Non-Ambulatory | 6 | 0.05 |
| Overall bad health status | 1 | 0.01 |
| Pain | 1 | 0.01 |
| Posture | 3 | 0.03 |
| Relief Limb | 6 | 0.05 |
| Severity Joint | 1 | 0.01 |
| Severity Lameness | 3 | 0.03 |
| Sitting (frog) | 2 | 0.02 |
| Skeleton injury | 2 | 0.02 |
| Skin injuries | 1 | 0.01 |
| Treatment Effect | 15 | 0.13 |
| Trembling | 1 | 0.01 |
| Tumor | 2 | 0.02 |
|  |  |  |
| **Total** | 117 | 1.04 |

##### Table F1.4: Summary of open reported “specific clinical aspects” for PD: umbilical outpouching

|  | **n** | **%** |
| --- | --- | --- |
| Apathy | 1 | 0.03 |
| Diagnosis | 8 | 0.22 |
| Increase outpouching | 2 | 0.06 |
| Injury outpouching | 9 | 0.25 |
| Non-transportable | 2 | 0.06 |
| Overall bad health status | 1 | 0.03 |
| Position outpouching (e.g. tarsal joint) | 6 | 0.17 |
| Retarded development | 1 | 0.03 |
| Size of outpouching | 5 | 0.14 |
| State injury | 1 | 0.03 |
|  |  |  |
| **Total** | 36 | 1.02 |

##### Table F1.5: Summary of open reported “specific clinical aspects” for PD: respiratory disorder

|  | **n** | **%** |
| --- | --- | --- |
| Dyspnea | 3 | 0.75 |
| Apathy | 1 | 0.25 |
|  |  |  |
| **Total** | 4 | 1 |

##### Table F1.6: Summary of open reported “specific clinical aspects” for PD: broken bone

|  | **n** | **%** |
| --- | --- | --- |
| Diagnosis | 22 | 0.92 |
| Dysfunction | 2 | 0.08 |
|  |  |  |
| **Total** | 24 | 1 |

##### Figure 1: Frequency of clinical aspects mentioned in at least four primary diagnosis “PD”

|  | CNS disorder | Locomotor tract disorder | Umbilical outpouching | Respiration tract disorder | Broken Bone |
| --- | --- | --- | --- | --- | --- |
| Treatment effect, PD:4 | 17 | 111 | 1 | 8 |  |
| Bad overall health status, PD:5 | 5 | 91 | 17 | 8 | 3 |
| Diagnoses, PD:5 | 14 | 43 | 16 | 3 | 33 |
| Low body condition, PD:5 | 9 | 54 | 10 | 9 | 4 |
| Retarded development, PD:4 |  | 34 | 3 | 7 | 2 |
| Apathy, PD:4 | 3 | 1 | 3 | 7 |  |
| Chronicity, PD:4 | 1 | 5 | 1 | 1 |  |

##### Figure 2: Specific reasons mentioned for at least two PDs

|  | CNS disorder | Locomotor tract disorder | Umbilical outpouching | Respiratory tract disorder | Broken Bone |
| --- | --- | --- | --- | --- | --- |
| Diagnosis, PD:4 | 1 | 19 | 8 |  | 22 |
| Treatment Effect, PD:2 | 2 | 15 |  |  |  |
| Non-ambulatory, PD:2 | 4 | 6 |  |  |  |
| NoFood, PD:2 |  | 1 |  |  | 2 |
| Apathy, PD:2 |  |  | 1 | 1 |  |

#### F2 Combination of clinical aspects

##### Table F2.1: Comparing the vote for “specific clinical aspects” per primary diagnosis

| *n*  *row pct*  *col pct* | **Primary diagnosis** | | | | | |
| --- | --- | --- | --- | --- | --- | --- |
|  | **Broken mandibular bone** | **Umbilical outpouching** | **CNS disorder** | **Locomotor tract** | **Respiratory disorder** | **Total**  **(n, pct)** |
| **Yes** | 2; 0.14; 0.54; 3.45 | 33; 2.27; 8.97; 27.27 | 28; 1.92; 7.61; 23.53 | 273; 18.75; 74.18; 26.33 | 32; 2.20; 8.70; 26.45 | 368; 25.27 |
| **No** | 56; 3.85; 5.15; 96.55 | 88; 6.04; 8.09; 72.73 | 91; 6.25; 8.36; 76.47 | 764; 52.47; 70.22; 73.67 | 89; 6.11; 8.18; 73.55 | 1088; 74.73 |
| **Total** | 58; 3.98 | 121; 8.31 | 119; 8.17 | 1037; 71.22 | 121; 8.31 | 1456; 100.00 |

**Statistics for Table F2.1: Chi²-value: 15.7722, p-value: 0.0033.**

##### Table F2.2: Combination of reasons for PD: CNS disorder

|  | **n** | **%** |
| --- | --- | --- |
| Apathy | 1 | 0.04 |
| Ataxia | 1 | 0.04 |
| Biting injury | 1 | 0.04 |
| Diagnosis | 6 | 0.21 |
| Loss Reflexes | 1 | 0.04 |
| Low Body Condition | 3 | 0.11 |
| Non-ambulatory | 5 | 0.18 |
| Overall bad health status | 1 | 0.04 |
| Paddling | 2 | 0.07 |
| Severity state | 1 | 0.04 |
| Temperature | 2 | 0.07 |
| Treatment effect | 4 | 0.14 |
|  |  |  |
| **Total** | 28 | 1.02 |

##### Table F2.3: Combination of reasons for Locomotor disorder

|  | **n** | **%** |
| --- | --- | --- |
| Additional diagnosis | 28 | 0.07 |
| Biting injuries | 2 | 0.01 |
| Coughing | 1 | 0 |
| Decubitus | 1 | 0 |
| Diagnosis | 5 | 0.01 |
| Diagnosis pathogen | 1 | 0 |
| Dolphin head | 1 | 0 |
| Dyspnoea | 5 | 0.01 |
| Fever | 22 | 0.06 |
| Hindlimb paresis / paralysis | 26 | 0.07 |
| Hunched back | 6 | 0.02 |
| Infection | 3 | 0.01 |
| Infection joint | 2 | 0.01 |
| Joint elastic swelling | 2 | 0.01 |
| Joint hard. Red | 7 | 0.02 |
| Lameness | 59 | 0.15 |
| Loss reflexes | 3 | 0.01 |
| Low body condition | 22 | 0.06 |
| Mouth breathing | 1 | 0 |
| No food and water | 6 | 0.02 |
| Non-ambulatory | 12 | 0.03 |
| Overal bad health status | 19 | 0.05 |
| Pain | 5 | 0.01 |
| Paresis / paralysis |  | 0 |
| Posture | 10 | 0.03 |
| Pumping breath | 1 | 0 |
| Reduced behavior | 2 | 0.01 |
| Relief limb | 24 | 0.06 |
| Retarded development | 17 | 0.04 |
| Severity injury | 4 | 0.01 |
| Severity lameness | 16 | 0.04 |
| Severity state | 3 | 0.01 |
| Severity tumor | 2 | 0.01 |
| Sitting (frog) | 2 | 0.01 |
| Skin injury | 2 | 0.01 |
| Slow getting up | 3 | 0.01 |
| State (injury) | 3 | 0.01 |
| Tail injury | 27 | 0.07 |
| Treatment effect | 24 | 0.06 |
| Trembling | 3 | 0.01 |
| Tumor | 17 | 0.04 |
|  |  |  |
| **Total** | 399 | 1.07 |

##### Table F2.4: Combination of reasons for umbilical outpouching

|  | **n** | **%** |
| --- | --- | --- |
| Additional diagnosis | 2 | 0.04 |
| Apathy | 2 | 0.04 |
| Fever | 2 | 0.04 |
| Diagnosis | 4 | 0.08 |
| Increase outpouching | 3 | 0.06 |
| Injury outpouching | 11 | 0.22 |
| Low body condition | 8 | 0.16 |
| Non-transportable | 2 | 0.04 |
| Overall bad health condition | 7 | 0.14 |
| Position outpouching tarsal joint | 1 | 0.02 |
| Reduced behavior | 1 | 0.02 |
| Retarded development | 1 | 0.02 |
| Size outpouching | 1 | 0.02 |
| State injury | 1 | 0.02 |
| Tail injury | 3 | 0.06 |
|  |  |  |
| **Total** | 49 | 0.98 |

##### Table F2.5: Combination of reasons for Respiratory tract

|  | **n** | **%** |
| --- | --- | --- |
| Additional diagnosis | 1 | 0.03 |
| Apathy | 1 | 0.03 |
| Coughing | 1 | 0.03 |
| Diagnosis | 1 | 0.03 |
| Dyspnoea | 6 | 0.2 |
| Low body condition | 3 | 0.1 |
| Mouth breathing | 3 | 0.1 |
| Nasal discharge | 1 | 0.03 |
| Overall bad health status | 1 | 0.03 |
| Pumping breath | 4 | 0.13 |
| Retarded development | 5 | 0.17 |
| Severity retarded development | 1 | 0.03 |
| Treatment effect | 2 | 0.07 |
|  |  |  |
| **Total** | 30 | 0.98 |

##### Table F2.6: Combination of reasons for Broken bone

|  | **n** | **%** |
| --- | --- | --- |
| Overall bad health | 1 | 0.125 |
| Diagnosis | 2 | 0.25 |
| Pain | 1 | 0.125 |
| No food | 2 | 0.25 |
| Low body condition | 1 | 0.125 |
| Retarded development | 1 | 0.125 |
|  |  |  |
| **Total** | 8 | 1.00 |

##### Figure 3: Combinations of reasons shared across three PDs

|  | CNS disorder | Locomotor tract disorder | Umbilical outpouching | Respiratory tract disorder | Broken Bone |
| --- | --- | --- | --- | --- | --- |
| Low Body Condition, PD:5 | 3 | 22 | 8 | 3 | 1 |
| Overal bad health status, PD:5 | 1 | 19 | 7 | 1 | 1 |
| Diagnosis, PD:5 | 6 | 5 | 4 | 1 | 2 |
| Retarded development, PD:4 |  | 17 | 1 | 5 | 1 |
| Additional diagnosis, PD:3 |  | 28 | 2 | 1 |  |
| Treatment effect, PD:3 | 4 | 24 |  | 2 |  |
| Temperature, PD:3 | 2 | 22 | 2 |  |  |
| Apathy, PD:3 | 1 |  | 2 | 1 |  |

#### F3 Severity of clinical aspects

##### Table F3.1: Comparing the vote for severity per primary diagnosis

| *n*  *row pct*  *col pct* | **Primary Diagnosis** | | | | | |
| --- | --- | --- | --- | --- | --- | --- |
|  | **Broken mandimbular bone** | **Umbilical outpouching** | **CNS disorder** | **Locomotor tract** | **Respiratory disorder** | **Total**  **(n, pct)** |
| **yes** | 13; 0.89; 2.95; 22.41 | 29; 1.99; 6.59; 23.97 | 39; 2.68; 8.86; 32.77 | 322; 22.12; 73.18; 31.05 | 37; 2.54; 8.41; 30.58 | 440; 30.22 |
| **no** | 45; 3.09; 4.43; 77.59 | 92; 6.32; 9.06; 76.03 | 80; 5.49; 7.87; 67.23 | 715; 49.11; 70.37; 68.95 | 84; 5.77; 8.27; 69.42 | 1016; 69.78 |
| **Total** | 58; 3.98 | 121; 8.31 | 119; 8.17 | 1037; 71.22 | 121; 8.31 | 1456; 100.00 |

**Statistic for Table: F3.1: CHI²-value: 4.6345, p-value: 0.327.**

##### Table F3.2: Severity for the PD CNS disorder

|  | **n** | **%** |
| --- | --- | --- |
| Apathy | 2 | 0.05 |
| Ataxia | 2 | 0.05 |
| Diagnosis | 5 | 0.13 |
| Diagnosis pathogen | 1 | 0.03 |
| Low body condition | 4 | 0.1 |
| No food and water | 1 | 0.03 |
| Non-ambulatory | 9 | 0.23 |
| Overall bad health status | 1 | 0.03 |
| Paddling | 2 | 0.05 |
| Reduced behavior | 1 | 0.03 |
| Severity state | 4 | 0.1 |
| Temperature | 3 | 0.08 |
| Treatment effect | 5 | 0.13 |
|  |  |  |
| **Total** | 40 | 1.04 |

##### Table F3.3: Severity for the PD locomotor disorder

|  | **n** | **%** |
| --- | --- | --- |
| Abnormal bristles | 2 | 0.01 |
| Additional diagnosis | 11 | 0.03 |
| Apathy | 1 | 0 |
| Biting injury | 2 | 0.01 |
| Chronicity | 1 | 0 |
| Diagnosis | 14 | 0.04 |
| Dyspnoea | 1 | 0 |
| Fever | 13 | 0.04 |
| Hindlimb paresis / paralysis | 30 | 0.08 |
| Hunched back | 5 | 0.01 |
| Infection | 4 | 0.01 |
| Joint | 2 | 0.01 |
| Joint hard. Red | 1 | 0 |
| Joint swelling fluctuating | 1 | 0 |
| Lameness | 53 | 0.14 |
| Loss reflexes | 1 | 0 |
| Low body condition | 19 | 0.05 |
| Mouth breathing | 3 | 0.01 |
| Nasal discharge | 1 | 0 |
| No food | 2 | 0.01 |
| Non-ambulatory | 11 | 0.03 |
| Overall bad health state | 18 | 0.05 |
| Pain | 10 | 0.03 |
| Posture | 24 | 0.07 |
| Pumping breath | 2 | 0.01 |
| Reduced behavior | 3 | 0.01 |
| Relief limb | 28 | 0.08 |
| Retarded development | 9 | 0.02 |
| Saber | 1 | 0 |
| Severity injury | 5 | 0.01 |
| Severity joints | 3 | 0.01 |
| Severity lameness | 22 | 0.06 |
| Severity pain | 1 | 0 |
| Severity state | 3 | 0.01 |
| Severity tumor | 1 | 0 |
| Sitting (frog) | 3 | 0.01 |
| Skin injury | 1 | 0 |
| Slow getting up | 1 | 0 |
| Suffering | 1 | 0 |
| Tail injury | 11 | 0.03 |
| Trembling | 2 | 0.01 |
| Tumor | 15 | 0.04 |
| Treatment effect | 25 | 0.07 |
|  |  |  |
| **Total** | 367 | 1 |

##### Table F3.4: Severity for the PD umbilical outpouching

|  | **n** | **%** |
| --- | --- | --- |
| Additional diagnosis | 1 | 0.03 |
| Fever | 2 | 0.06 |
| Diagnosis | 3 | 0.1 |
| Increase outpouching | 3 | 0.1 |
| Injury outpouching | 5 | 0.16 |
| Low body condition | 2 | 0.06 |
| Non-transportable | 2 | 0.06 |
| Overall bad health status | 2 | 0.06 |
| Position outpouching (eg tarsal joint) | 3 | 0.1 |
| size outpouching | 3 | 0.1 |
| State injury | 3 | 0.1 |
| Tail injury | 1 | 0.03 |
| Treatment effect | 1 | 0.03 |
|  |  |  |
| **Total** | 31 | 0.99 |

##### Table F3.5: Severity for respiratory tract

|  | **n** | **%** |
| --- | --- | --- |
| Additional diagnosis | 4 | 0.1 |
| Apathy | 5 | 0.12 |
| Diagnosis | 2 | 0.05 |
| Dyspnoea | 10 | 0.24 |
| Exopthalmus | 2 | 0.05 |
| Low body condition | 4 | 0.1 |
| Mouth breathing | 5 | 0.12 |
| Overall bad health status | 1 | 0.02 |
| Pale | 1 | 0.02 |
| Pumpung breath | 3 | 0.07 |
| Retarded development | 2 | 0.05 |
| Treatment effect | 2 | 0.05 |
|  |  |  |
| **Total** | 41 | 0.99 |

##### Table F3.6: Severity for suspected broken bone

|  | **n** | **%** |
| --- | --- | --- |
| Diagnosis | 8 | 0.47 |
| No food | 5 | 0.29 |
| Pain | 2 | 0.12 |
| Low body condition | 2 | 0.12 |
|  |  |  |
| **Total** | 17 | 1 |

##### Figure 4: Clinical signs characterizing the severity shared across PDs

|  | CNS disorder | Locomotor tract disorder | Umbilical Outpouching | Respiratory tract disorder | Broken Bone |
| --- | --- | --- | --- | --- | --- |
| Diagnosis, PD:5 | 5 | 14 | 3 | 2 | 8 |
| Low Body Condition, PD:5 | 4 | 19 | 2 | 4 | 2 |
| Treatment Effect, PD:4 | 5 | 25 | 1 | 2 |  |
| Overall bad health state, PD:4 | 1 | 18 | 2 | 1 |  |

#### F4 Overall health state

##### Table F4.1: Comparing the vote for overall health state per primary diagnosis

| *n*  *row pct*  *col pct* | **Primary Diagnosis** | | | | | |
| --- | --- | --- | --- | --- | --- | --- |
|  | **Broken mandimbular bone** | **Umbilical outpouching** | **CNS disorder** | **Locomotor tract** | **Respiratory disorder** | **Sum**  **(n, pct)** |
| **Yes** | 4; 0.27; 1.13; 6.90 | 24; 1.65; 6.76; 19.83 | 35; 2.40; 9.86; 29.41 | 254; 17.45; 71.55; 24.49 | 38; 2.61; 10.70; 31.40 | 355; 24.38 |
| **No** | 54; 3.71; 4.90; 93.10 | 97; 6.66; 8.81; 80.17 | 84; 5.77; 7.63; 70.59 | 783; 53.78; 71.12; 75.51 | 83; 5.70; 7.54; 68.60 | 1101; 75.62 |
| **Sum** | 58; 3.98 | 121; 8.31 | 119; 8.17 | 1037; 71.22 | 121; 8.31 | 1456; 100.00 |

**Statistics for Table F4.1: CHI²-value: 15.8519, p-value: 0.0032.**

##### Table F4.2: Overall state of suspected CNS disorder

|  | **n** | **%** |
| --- | --- | --- |
| Acuteness | 2 | 0.07 |
| Apathy | 2 | 0.07 |
| Ataxia | 1 | 0.03 |
| Chronicity | 1 | 0.03 |
| Diagnosis | 2 | 0.07 |
| Low Body Condition | 2 | 0.07 |
| No food and water | 3 | 0.1 |
| Non-Ambulatory | 3 | 0.1 |
| Overall bad health state | 3 | 0.1 |
| Severity state | 3 | 0.1 |
| Temperature | 2 | 0.07 |
| Treatment Effect | 6 | 0.2 |
|  |  |  |
| **Total** | 30 | 1.01 |

##### Table F4.3: Overall state of suspected locomotor disease

|  | **n** | **%** |
| --- | --- | --- |
| Abnormal bristles | 8 | 0.03 |
| Acuteness | 8 | 0.03 |
| Additional diagnosis | 10 | 0.03 |
| Apathy | 3 | 0.01 |
| Biting injury | 6 | 0.02 |
| Chronicity | 4 | 0.01 |
| Diagnosis | 5 | 0.02 |
| Dyspnea | 1 | 0 |
| Fever | 8 | 0.03 |
| Hindlimb paresis/paralysis | 5 | 0.02 |
| Hunched back | 3 | 0.01 |
| Infection | 3 | 0.01 |
| Lameness | 25 | 0.09 |
| Loss reflexes | 1 | 0 |
| Low body condition | 13 | 0.05 |
| Nasal discharge | 1 | 0 |
| No food | 3 | 0.01 |
| Non-ambulatory | 9 | 0.03 |
| Non-transportable | 1 | 0 |
| Overall bad health status | 53 | 0.18 |
| Pain | 12 | 0.04 |
| Pale | 1 | 0 |
| Posture | 2 | 0.01 |
| Reduced behavior | 2 | 0.01 |
| Relief limb | 2 | 0.01 |
| Retarded development | 8 | 0.03 |
| Severity injury | 3 | 0.01 |
| Severity lameness | 8 | 0.03 |
| Severity pain | 4 | 0.01 |
| Severity state | 10 | 0.03 |
| Sitting (frog) | 1 | 0 |
| Suffering | 7 | 0.02 |
| Tail injury | 1 | 0 |
| Treatment effect | 47 | 0.16 |
| Tumor | 10 | 0.03 |
|  |  |  |
| **Total** | 288 | 0.96 |

##### Table F4.4: Overall state of suspected umbilical outchpouching

|  | n | % |
| --- | --- | --- |
| Biting injuries | 1 | 0.05 |
| Chronicity | 1 | 0.05 |
| Diagnosis | 1 | 0.05 |
| Increase outpouching | 3 | 0.14 |
| Injury outpouching | 3 | 0.14 |
| Non-transportable | 2 | 0.09 |
| Overall bad health status | 7 | 0.32 |
| Retarded development | 1 | 0.05 |
| Size outpouching | 2 | 0.09 |
| Tail injury | 1 | 0.05 |
|  |  |  |
| **Total** | **22** | **1.03** |

##### Table F4.5: Overall state of suspected respiratory tract

|  | **n** | **%** |
| --- | --- | --- |
| Abnormal bristles | 2 | 0.10 |
| Additional diagnosis | 1 | 0.05 |
| Apathy | 1 | 0.05 |
| Chronicity | 1 | 0.05 |
| Dyspnoea | 3 | 0.14 |
| Low body condition | 2 | 0.10 |
| Mouth breathing | 1 | 0.05 |
| Overall bad health state | 6 | 0.29 |
| Treatment effect | 4 | 0.19 |
|  |  |  |
| **Total** | 21 | 1.02 |

##### Table F4.6: Overall state of suspected broken bone

|  | **n** | **%** |
| --- | --- | --- |
| Overall bad health status | 2 | 0.25 |
| Diagnosis | 1 | 0.13 |
| Pain | 1 | 0.13 |
| No food | 1 | 0.13 |
| Low body condition | 1 | 0.13 |
| Retarded development | 1 | 0.13 |
| Acuteness | 1 | 0.13 |
|  |  |  |
| **Total** | 8 | 1.03 |

##### Figure 5: Overall state shared overall across PDs

|  | Locomotor tract | RST | fracture | umbilical hernia | susp cns |  |
| --- | --- | --- | --- | --- | --- | --- |
| Overall bad health state, pd:5 | 53 | 6 | 2 | 7 | 3 | 71 |
| low body condition, pd:4 | 13 | 2 | 1 |  | 2 | 18 |
| chronicity, pd:4 | 4 | 1 |  | 1 | 1 | 7 |
| Diagnosis, pd:4 | 5 |  | 1 | 1 | 2 | 9 |
| Treatment effect, pd:3 | 47 | 4 |  |  | 6 | 57 |
| acuteness, pd:3 | 8 |  | 1 |  | 2 | 11 |
| Retarded development, pd:3 | 8 |  | 1 | 1 |  | 10 |
| NoFood, pd:3 | 3 |  | 1 |  | 3 | 7 |

#### F5 Treatment chances

##### Table F5.1: Comparing the vote for “specific clinical aspects” per primary diagnosis

| *n*  *row pct*  *col pct* | **Primary diagnosis** | | | | | |
| --- | --- | --- | --- | --- | --- | --- |
|  | **Broken mandibular bone** | **Umbilical outpouching** | **CNS disorder** | **Locomotor tract** | **Respiratory disorder** | **Sum**  **(n, pct)** |
| **Yes** | 27; 1.85; 4.55; 46.55 | 27; 1.85; 4.55; 22.31 | 50; 3.43; 8.42; 42.02 | 450; 30.91; 75.76; 43.39 | 40; 2.75; 6.73; 33.06 | 594; 40.80 |
| **No** | 31; 2.13; 3.60; 53.45 | 94; 6.46; 10.90; 77.69 | 69; 4.74; 8.00; 57.98 | 587; 40.32; 68.10; 56.61 | 81; 5.56; 9.40; 66.94 | 862; 59.20 |
| **Sum** | 58; 3.98 | 121; 8.31 | 119; 8.17 | 1037; 71.22 | 121; 8.31 | 1456; 100.00 |

**Statistics for Table 5.1.: CHI²-value: 23.8799, p-value: <.0001.**

##### Table F5.2: Treatment chances per PD in comparison

| *n*  *row pct*  *col pct* | **Primary diagnosis** | | | | | |
| --- | --- | --- | --- | --- | --- | --- |
|  | **Broken mandibular bone** | **Umbilical outpouching** | **CNS disorder** | **Locomotor tract** | **Respiratory disorder** | **Total**  **(n, pct)** |
| **Other response** | 1; 0.30; 11.11; 4.00 | 1; 0.30; 11.11; 7.69 | 1; 0.30; 11.11; 3.85 | 6; 1.78; 66.67; 2.39 | 0; 0.00; 0.00; 0.00 | 9; 2.67 |
| **Good** | 0; 0.00; 0.00; 0.00 | 0; 0.00; 0.00; 0.00 | 0; 0.00; 0.00; 0.00 | 1; 0.30; 100.00; 0.40 | 0; 0.00; 0.00; 0.00 | 1; 0.30 |
| **Grave** | 22; 6.53; 8.15; 88.00 | 12; 3.56; 4.44; 92.31 | 21; 6.23; 7.78; 80.77 | 197; 58.46; 72.96; 78.49 | 18; 5.34; 6.67; 81.82 | 270; 80.12 |
| **Moderate** | 2; 0.59; 3.51; 8.00 | 0; 0.00; 0.00; 0.00 | 4; 1.19; 7.02; 15.38 | 47; 13.95; 82.46; 18.73 | 4; 1.19; 7.02; 18.18 | 57; 16.91 |
| **Total** | 25; 7.42 | 13; 3.86 | 26; 7.72 | 251; 74.48 | 22; 6.53 | 337; 100.00 |
| Missing = 413 | | | | | | |

**Statistic for Table F5.2: CHI²-value: 6.9761, p-value: 0.8592.**

#### F6 Time-frame

##### Table F6.1: Comparing the vote for “time-frame” per primary diagnosis

| *n*  *row pct*  *col pct* | **Primary diagnosis** | | | | | |
| --- | --- | --- | --- | --- | --- | --- |
|  | **Broken mandibular bone** | **Umbilical outpouching** | **CNS disorder** | **Locomotor tract** | **Respiratory disorder** | **Sum**  **(n, pct)** |
| **Yes** | 0; 0.00; 0.00; 0.00 | 12; 0.82; 3.54; 9.92 | 44; 3.02; 12.98; 36.97 | 235; 16.14; 69.32; 22.66 | 48; 3.30; 14.16; 39.67 | 339; 23.28 |
| **No** | 58; 3.98; 5.19; 100.00 | 109; 7.49; 9.76; 90.08 | 75; 5.15; 6.71; 63.03 | 802; 55.08; 71.80; 77.34 | 73; 5.01; 6.54; 60.33 | 1117; 76.72 |
| **Sum** | 58; 3.98 | 121; 8.31 | 119; 8.17 | 1037; 71.22 | 121; 8.31 | 1456; 100.00 |

**Statistic for Table 5.2: CHI²-value: 60.6071, p-value: <.0001.**

##### Table F6.2: Time until considered more critical

| **n** | **Primary diagnosis** | | | | | |
| --- | --- | --- | --- | --- | --- | --- |
|  | **Broken mandibular bone** | **Umbilical outpouching** | **CNS disorder** | **Locomotor tract** | **Respiratory disorder** | **Sum**  **(n, pct)** |
| **1 day** | 0 | 0 | 0 | 9 | 0 | 9 (13.85) |
| **2 days** | 0 | 0 | 4 | 12 | 0 | 16 (24.62) |
| **3 days** | 0 | 1 | 2 | 17 | 1 | 21 (32.31) |
| **4 - 7 days** | 0 | 2 | 1 | 7 | 1 | 11 (16.92) |
| **8 - 14 days** | 0 | 0 | 0 | 6 | 0 | 6 (9.23) |
| **Non critical** | 0 | 1 | 0 | 1 | 0 | 2 (3.08) |
| **Total** | 0 | 4 | 7 | 52 | 2 | 65 |
| **missing = 1391** | | | | | | |

**Statistics for table F5.2: CHI²-value: 19.6042, p-value: 0.1876.**

## G Comparing reported reasons regarding professions

### Table G1: Chance for treatment success comparing professions

| *n*  *pct*  *row pct*  *col pct* | **Profession** | | |
| --- | --- | --- | --- |
|  | **Farmer** | **Veterinarian** | **Sum** |
| **Yes** | 67; 4.60; 11.28; 29.91 | 527; 36.20; 88.72; 42.78 | 594; 40.80 |
| **No** | 157; 10.78; 18.21; 70.09 | 705; 48.42; 81.79; 57.22 | 862; 59.20 |
| **Total** | 224; 15.38 | 1232; 84.62 | 1456; 100.00 |

**Statistics for Table G1: Fisher’s Exact Test (two sided), p-value: 0.0003.**

### Table G2: Severity comparing professions

| *n*  *pct*  *row pct*  *col pct* | **Profession** | | |
| --- | --- | --- | --- |
|  | **Farmer** | **Veterinarians** | **Total** |
| **Yes** | 37; 2.54; 8.41; 16.52 | 403; 27.68; 91.59; 32.71 | 440; 30.22 |
| **No** | 187; 12.84; 18.41; 83.48 | 829; 56.94; 81.59; 67.29 | 1016; 69.78 |
| Sum | 224; 15.38 | 1232; 84.62 | 1456; 100.00 |

**Statistic for Table G2: Fisher’s Exact Test (two sided) p-value <.0001.**

### Table G3: Combination of symptoms comparing veterinary professions

| *n pct*  *row pct*  *col pct* | **Profession** | | |
| --- | --- | --- | --- |
|  | **Veterinarian (public official)** | **Veterinarian (practicing)** | **SUM** |
| **Yes** | 45; 3.88; 26.16; 9.89 | 127; 10.95; 73.84; 18.01 | 172; 14.83 |
| **No** | 410; 35.34; 41.50; 90.11 | 578; 49.83; 58.50; 81.99 | 988; 85.17 |
| **Total** | 455; 39.22 | 705; 60.78 | 1160; 100.00 |

**Statistics for Table G3: Fisher’s Exact Test (two-sided), p-value: 0.0001.**

### Table G4: Severity of symptoms comparing veterinarians’ profession

| *n*  *pct*  *row pct*  *col pct* | **Veterinarians‘ Profession** | | |
| --- | --- | --- | --- |
|  | **Public official** | **Practicing** | **Total** |
| **Yes** | 98; 8.45; 32.34; 21.54 | 205; 17.67; 67.66; 29.08 | 303; 26.12 |
| **No** | 357; 30.78; 41.66; 78.46 | 500; 43.10; 58.34; 70.92 | 857; 73.88 |
| **Total** | 455; 39.22 | 705; 60.78 | 1160; 100.00 |

**Statistics for Table G4, Fisher’s Exact Test (two sided): 0.0050.**

### Table G5: Overall health state comparing veterinarians’ profession

|  | **Profession** | | |
| --- | --- | --- | --- |
|  | **Public official** | **Practicing** | **Total** |
| **Yes** | 137; 11.81; 46.13; 30.11 | 160; 13.79; 53.87; 22.70 | 297; 25.60 |
| **No** | 318; 27.41; 36.85; 69.89 | 545; 46.98; 63.15; 77.30 | 863; 74.40 |
| **Total** | 455; 39.22 | 705; 60.78 | 1160; 100.00 |

**Statistics for Table G4, Fisher’s Exact Test (two sided): 0.0058.**

### Table G6: Overall health state comparing veterinarians’ profession

|  | **Veterinarian profession** | | |
| --- | --- | --- | --- |
|  | **Public official** | **Practicing** | **Total** |
| **Yes** | 176; 15.17; 36.44; 38.68 | 307; 26.47; 63.56; 43.55 | 483; 41.64 |
| **No** | 279; 24.05; 41.21; 61.32 | 398; 34.31; 58.79; 56.45 | 677; 58.36 |
| **Total** | 455; 39.22 | 705; 60.78 | 1160; 100.00 |

**Statistics for G6, Fisher’s Exact Test (two sided): 0.1127.**

### Table G7: Overall health state comparing veterinarians’ profession

| **Duration of sickness** | **Veterinarian profession** | | |
| --- | --- | --- | --- |
|  | **Public official** | **Practicing** | **Total** |
| **Yes** | 93; 8.02; 35.23; 20.44 | 171; 14.74; 64.77; 24.26 | 264; 22.76 |
| **No** | 362; 31.21; 40.40; 79.56 | 534; 46.03; 59.60; 75.74 | 896; 77.24 |
| **Total** | 455; 39.22 | 705; 60.78 | 1160; 100.00 |

**Statistics for Table G7, Fisher’s Exact Test: 0.1328.**

## H Comparing cases of the evaluation and overall data set, qualified majority

##### Table H1.1: Variables characterizing impaired overall health state

| **Clinical sign** | % total  *col pct* | **Qualified euthanized (QE)** | **Qualified recovered (QR)** | **Unclear cases**  **(UC)** | **Total (n)** | **CHI²-value** | **p-value** |
| --- | --- | --- | --- | --- | --- | --- | --- |
| Bad overall health condition | nr | *6.95*  *32.9* | 33.4  *67.2* | 10.2  *34.9* | 189 | 41.2 | <.0001 |
|  | bad | 14.2  *67.1* | 16.3  *32.8* | 19  *65.1* | 185 |  |  |
| Abnormal temperature | nr | 9.0  *42.7* | 31.64  *62.6* | 15.5  *55* | 199 | 8.6 | 0.014 |
|  | abnormal | 12.2  *57.3* | 18.9  37.4 | 12.7  *45* | 155 |  |  |
| Abnormal behavior | nr | 14.1  *66.2* | 42.1  *85.9* | 22.2  *25.2* | 283 | 13.4 | 0.0012 |
|  | abnormal | 7.2  *33.8* | 6.9  *14.1* | 7.5  *25.2* | 78 |  |  |
| Low body condition | nr | 13.3  *63.3* | 41.2  *82.5* | 17  *28.7* | 269 | 22.4 | <.0001 |
|  | low | 7.7  *36.7* | 8.8  *17.6* | 12  *41.3* | 107 |  |  |
| Retarded development  (compared to pen mates) | nr | 13.3  *63.3* | 41.0  *82* | 19.4  *67.0* | 277 | 13.5 | 0.0012 |
|  | retarded | 7.7  *36.7* | 9.0  *18.1* | 9.6  *33.0* | 99 |  |  |
| Long bristles | nr | 13.2  *67.7* | 39.7  *85.2* | 22.4  *66.1* | 131 | 8.01 | 0.018 |
|  | long | 6.3  *32.4* | 6.9  *14.8* | 11.5  *33.9* | 43 |  |  |
| Difficulties accessing food | nr | 19.7  *93.7* | 46.5  *93.1* | 24.7  *85.5* | 342 | 5.95 | 0.051 |
|  | difficulties | 1.3  *6.3* | 3.5  *7.0* | 4.3  *14.7* | 34 |  |  |
| Total n = absolute frequency; % total: percentage of absolute frequency; col pct = column percent  nr = non remarkable | | | | | | | |

##### Table H1.2: Variables characterizing impaired posture

| **Clinical sign** | % total  *col pct* | **Qualified euthanized (QE)** | **Qualified recovered (QR)** | **Unclear cases**  **(UC)** | **Total (n)** | **CHI²-value** | **p-value** |
| --- | --- | --- | --- | --- | --- | --- | --- |
| Carping* | nr | 20.5  *97.5* | 50  *100.0* | 29  *100.0* | 374 | 7.76 | 0.023 |
|  | carping | 0.5  *2.5* | 0  *0.0* | 0  *0.0* | 2 |  |  |
| Difficulty rising | nr | 9.3  *30.6* | 18.6  *62.9* | 22.9  *57.5* | 60 | 15.18 | 0.0044 |
|  | difficulty | 15.3  *50.0* | 11  *37.1* | 15.3  *38.3* | 49 |  |  |
|  | impossible | 5.9  *19.4* | 0  *0* | 1.7  *4.3* | 9 |  |  |
| Hunched back | nr | 17  *81.0* | 44.5  *89.4* | 20.7  *71.6* | 310 | 15.25 | 0.0005 |
|  | hunched | 4  *19.0* | 5.3  *10.6* | 8.2  *28.4* | 66 |  |  |
| *carping defines the weight bearing on both frontal limbs or by knuckling  total n = absolute frequency; % total: percentage of absolute frequency; col pct = column percent  nr= non remarkable | | | | | | | |

##### Table H1.3: Variables characterizing impaired locomotion

| **Clinical sign** | % total  *col pct* | **Qualified euthanized (QE)** | **Qualified recovered (QR)** | **Unclear cases**  **(UC)** | **Total (n)** | **CHI²-value** | **p-value** |
| --- | --- | --- | --- | --- | --- | --- | --- |
| Abnormal gait | nr | 20.7  *98.7* | 49.7  *99.5* | 28.2  *97.3* | 371 | 2.6 | 0.273 |
|  | abnormal | 0.3  *1.3* | 0.3  *0.5* | 0.8  *2.8* | 6 |  |  |
| Relief of limb | nr | 11.7  *55.7* | 39.6  *79.3* | 18.6  *64.2* | 263 | 17.08 | .0002 |
|  | relief | 9.3  *44.3* | 10.4  *20.4* | 10.4  *35.8* | 113 |  |  |
| Phase of affected locomotion | only when moving | 2.44  *9.5* | 9.78  *27.6* | 15.24  *39.1* | 45 | 21.93 | 0.0002 |
|  | when moving and standing | 16.5  *64.3* | 23.8  *67.2* | 14.6  *37.5* | 90 |  |  |
|  | no weight bearing | 6.7  *26.2* | 1.8  *5.2* | 9.2  *23.4* | 164 |  |  |
| Non-ambulatory* | nr | 20  *94.9* | 49.5  *98.9* | 29  *100.0* | 370 | 8.16 | 0.017 |
|  | non-ambulatory | 1.1  *5.1* | 0.5  *1.1* | 0  *0.0* | 6 |  |  |
| Hindlimb paresis/ paralysis | nr | 19.4  *92.4* | 50  *100* | 28.5  *98.2* | 368 | 15.472 | 0.0004 |
|  | Paresis / paralysis | 1.6  *7.6* | 0  *0* | 0.5  *1.8* | 8 |  |  |
| Joint enlarged | nr | 17.6  *83.5* | 39.4  *78.7* | 24.5  *84.4* | 306 | 1.78 | 0.411 |
|  | Enlarged | 3.5  *16.5* | 10.6  *21.3* | 4.52  *15.6* | 70 |  |  |
| Claw lesions | nr | 20.1  *95.1* | 29.4  *72.2* | 36.6  *96* | 167 | 21.6 | <.0001 |
|  | lesion | 1.0  *4.9* | 11.3  *27.9* | 1.6  *4.1* | 27 |  |  |
| *non-ambulatory = a pig cannot hold stance and or is not able to move without help over 15 seconds  total n = absolute frequency % total: percentage of absolute frequency; col pct = column percent  nr= non remarkable | | | | | | | |

##### Table H4: Variables characterizing specific disorders

| **Clinical sign** | % total  *col pct* | **Qualified euthanized (QE)** | **Qualified recovered (QR)** | **Unclear cases**  **(UC)** | **Total (n)** | **CHI²-value** | **p-value** |
| --- | --- | --- | --- | --- | --- | --- | --- |
| Umbilical outpouching | nr | 15.8  *77.4* | 40.8  *93.9* | 34.9  *96.4* | 139 | 10.02 | 0.007 |
|  | outpouching | 4.6  *22.6* | 2.63  *6.1* | 1.3  *3.6* | 13 |  |  |
| Size of umbilical outpouching *(ventral aspect is … )* | higher than tarsal joint | 15.4  *28.6* | 7.7  *25* | 0  *0* | 3 | 5.08 | 0.28 |
|  | equal to tarsal joint | 15.4  *28.6* | 23.1  *75* | 15.4  *100* | 7 |  |  |
|  | lower than tarsal joint | 23.1  *42.9* | 0  *0* | 0  *0* | 3 |  |  |
| Tail biting lesions | nr | 11.4  *44* | 28.5  *66.3* | 22.8  *73.3* | 121 | 10.83 | 0.0045 |
|  | lesion | 14.5  *56* | 14.5  *33.7* | 83  *26.7* | 72 |  |  |
| Loss of tail substance | nr | 5.1  *25* | 15.3  *30* | 10.8  *37.6* | 110 | 29.292 | <.0001 |
|  | no / small tissue loss | 1.1  *5.6* | 0.3  *0.6* | 1.7  *5.9* | 11 |  |  |
|  | medium to high grade | 6  *29.2* | 5.7  *11.11* | 2.3  *7.9* | 49 |  |  |
| Dyspnea | nr | 20.2  *96.2* | 48.1  *96.3* | 28.2  *97.3* | 363 | 0.23 | 0.892 |
|  | dyspnea | 0.8  *3.8* | 1.86  *3.72* | 0.8  *2.8* | 13 |  |  |
| total n = absolute frequency % total: percentage of absolute frequency; col pct = column percent  nr = non remarkable | | | | | | | |

## I Comparing cases of the evaluation and overall data set, qualified majority

| Variable | Categories | Clinical signs of pigs on the  decision day of euthanasia | | | Statistical assessment | |
| --- | --- | --- | --- | --- | --- | --- |
|  | Frequency Percent Row Pct Col Pct | Project Veterinarians | Evaluators | Total | CHI²-value | p-value  (or Fisher’s Exact Test) |
| Bad overall health status | Non remarkable | 1 2.50 33.33 5.00 | 2 5.00 66.67 10.00 | 3 7.50 | 1.0000 | |
|  | Abnormal | 19 47.50 51.35 95.00 | 18 45.00 48.65 90.00 | 37 92.50 |  |  |
|  | Total | 20 50.00 | 20 50.00 | 40 100.00 |  |  |
| Behavior | Apathy | 8 20.00 50.00 40.00 | 8 20.00 50.00 40.00 | 16 40.00 | 0.2588 | 0.9676 |
|  | unclear | 2 5.00 40.00 10.00 | 3 7.50 60.00 15.00 | 5 12.50 |  |  |
|  | reduced | 1 2.50 50.00 5.00 | 1 2.50 50.00 5.00 | 2 5.00 |  |  |
|  |  | 9 22.50 52.94 45.00 | 8 20.00 47.06 40.00 | 17 42.50 |  |  |
|  | Total | 20 50.00 | 20 50.00 | 40 100.00 |  |  |
| Retarded development | Non remarkable | 9 22.50 47.37 45.00 | 10 25.00 52.63 50.00 | 19 47.50 | 1.0000 | |
|  | Retarded development | 11 27.50 52.38 55.00 | 10 25.00 47.62 50.00 | 21 52.50 |  |  |
|  | Total | 20 50.00 | 20 50.00 | 40 100.00 |  |  |
| Lower body condition | Non remarkable | 9 22.50 50.00 45.00 | 9 22.50 50.00 45.00 | 18 45.00 | 1.0000 | |
|  | Low body condition | 11 27.50 50.00 55.00 | 11 27.50 50.00 55.00 | 22 55.00 |  |  |
|  | Total | 20 50.00 | 20 50.00 | 40 100.00 |  |  |
| Abnormal temperature | Non remarkable | 8 21.62 50.00 44.44 | 8 21.62 50.00 42.11 | 16 43.24 | 1.0000 | |
|  | Remarkable | 10 27.03 47.62 55.56 | 11 29.73 52.38 57.89 | 21 56.76 |  |  |
|  | Total | 18 48.65 | 19 51.35 | 37 100.00 |  |  |
| Coughing | Non remarkable | 1 16.67 25.00 50.00 | 3 50.00 75.00 75.00 | 4 66.67 | 1.0000 | |
|  | Coughing | 1 16.67 50.00 50.00 | 1 16.67 50.00 25.00 | 2 33.33 |  |  |
|  | Total | 2 33.33 | 4 66.67 | 6 100.00 |  |  |
| Abnormal bristles | Non remarkable | 5 26.32 41.67 55.56 | 7 36.84 58.33 70.00 | 12 63.16 | 0.6499 | |
|  | Abnormal bristles | 4 21.05 57.14 44.44 | 3 15.79 42.86 30.00 | 7 36.84 |  |  |
|  |  | 9 47.37 | 10 52.63 | 19 100.00 |  |  |
| Abnormal skin colour | Non remarkable | 9 47.37 52.94 100.00 | 8 42.11 47.06 80.00 | 17 89.47 | 0.4737 | |
|  | Abnormal skin colour | 0 0.00 0.00 0.00 | 2 10.53 100.00 20.00 | 2 10.53 |  |  |
|  |  | 9 47.37 | 10 52.63 | 19 100.00 |  |  |
| Skin injuries | Non remarkable | 6 31.58 46.15 75.00 | 7 36.84 53.85 63.64 | 13 68.42 |  | 1.0000 |
|  | Skin injuries | 2 10.53 33.33 25.00 | 4 21.05 66.67 36.36 | 6 31.58 |  |  |
|  | Total | 8 42.11 | 11 57.89 | 19 100.00 |  |  |
| Tail lesion | Non remarkable | 4 17.39 40.00 40.00 | 6 26.09 60.00 46.15 | 10 43.48 |  | 1.0000 |
|  | Lesion | 6 26.09 46.15 60.00 | 7 30.43 53.85 53.85 | 13 56.52 |  |  |
|  | Total | 10 43.48 | 13 56.52 | 23 100.00 |  |  |
| Loss of substance (tail) | Without loss | 1 7.69 33.33 16.67 | 2 15.38 66.67 28.57 | 3 23.08 | 1.3757 | 0.5027 |
|  | Small to medium loss | 4 30.77 44.44 66.67 | 5 38.46 55.56 71.43 | 9 69.23 |  |  |
|  | High loss | 1 7.69 100.00 16.67 | 0 0.00 0.00 0.00 | 1 7.69 |  |  |
|  | Total | 6 46.15 | 7 53.85 | 13 100.00 |  |  |
| Phase of lameness | When resting | 4 19.05 44.44 40.00 | 5 23.81 55.56 45.45 | 9 42.86 | 1.0000 | |
|  | No weight bearing on the limb | 6 28.57 50.00 60.00 | 6 28.57 50.00 54.55 | 12 57.14 |  |  |
|  | Total | 10 47.62 | 11 52.38 | 21 100.00 |  |  |
| Joint affected | Non remarkable | 4 14.81 44.44 30.77 | 5 18.52 55.56 35.71 | 9 33.33 | 1.0000 | |
|  | Increased size / increased filling | 9 33.33 50.00 69.23 | 9 33.33 50.00 64.29 | 18 66.67 |  |  |
|  | Total | 13 48.15 | 14 51.85 | 27 100.00 |  |  |
| Number of affected joints | Non remarkable | 11 27.50 50.00 55.00 | 11 27.50 50.00 55.00 | 22 55.00 | 0.2769 | 0.8707 |
|  | One limb | 6 15.00 46.15 30.00 | 7 17.50 53.85 35.00 | 13 32.50 |  |  |
|  | More than one limb | 3 7.50 60.00 15.00 | 2 5.00 40.00 10.00 | 5 12.50 |  |  |
|  | Total | 20 50.00 | 20 50.00 | 40 100.00 |  |  |
| Difficulty rising | Non remarkable | 1 4.55 33.33 9.09 | 2 9.09 66.67 18.18 | 3 13.64 | 1.0000 | |
|  | Difficult | 10 45.45 52.63 90.91 | 9 40.91 47.37 81.82 | 19 86.36 |  |  |
|  | Total | 11 50.00 | 11 50.00 | 22 100.00 |  |  |
| Prolapsus of the rectum | No | 4 50.00 57.14 80.00 | 3 37.50 42.86 100.00 | 7 87.50 | 1.0000 | |
|  | Yes | 1 12.50 100.00 20.00 | 0 0.00 0.00 0.00 | 1 12.50 |  |  |
|  | Total | 5 62.50 | 3 37.50 | 8 100.00 |  |  |

## J Comparing cases of the evaluation and overall data set, simple majority

| Variable | Categories | Clinical signs of pigs on the  decision day of euthanasia | | | Statistical assessment | | |
| --- | --- | --- | --- | --- | --- | --- | --- |
|  | Frequency Percent Row Pct Col Pct | Project Veterinarians | Evaluators | Total | CHI²-value | p-value  (or Fisher’s Exact Test) | |
| Bad overall health status | Non remarkable | 4 9.52 80.00 19.05 | 1 2.38 20.00 4.76 | 5 11.90 | 0.3433 | | |
|  | Abnormal | 17 40.48 45.95 80.95 | 20 47.62 54.05 95.24 | 37 88.10 |  |  |  |
|  | Total | 21 50.00 | 21 50.00 | 42 100.00 |  |  |  |
| Behavior | Non remarkable | 11 26.19 44.00 52.38 | 14 33.33 56.00 66.67 | 25 59.52 | 1.7703 | 0.6214 | |
|  | Apathy | 2 4.76 66.67 9.52 | 1 2.38 33.33 4.76 | 3 7.14 |  |  |  |
|  | unclear | 1 2.38 100.00 4.76 | 0 0.00 0.00 0.00 | 1 2.38 |  |  |  |
|  | reduced | 7 16.67 53.85 33.33 | 6 14.29 46.15 28.57 | 13 30.95 |  |  |  |
|  | Total | 21 50.00 | 21 50.00 | 42 100.00 |  |  |  |
| Retarded development | Non remarkable | 13 30.95 50.00 61.90 | 13 30.95 50.00 61.90 | 26 61.90 | 1.0000 | | |
|  | Retarded development | 8 19.05 50.00 38.10 | 8 19.05 50.00 38.10 | 16 38.10 |  |  |  |
|  | Total | 21 50.00 | 21 50.00 | 42 100.00 |  |  |  |
| Lower body condition | Non remarkable | 11 26.19 45.83 52.38 | 13 30.95 54.17 61.90 | 24 57.14 | 0.7557 | | |
|  | Low body condition | 10 23.81 55.56 47.62 | 8 19.05 44.44 38.10 | 18 42.86 |  |  |  |
|  | Total | 21 50.00 | 21 50.00 | 42 100.00 |  |  |  |
| Abnormal temperature | Non remarkable | 5 13.16 45.45 26.32 | 6 15.79 54.55 31.58 | 11 28.95 | 1.0000 | | |
|  | Remarkable | 14 36.84 51.85 73.68 | 13 34.21 48.15 68.42 | 27 71.05 |  |  |  |
|  | Total | 19 50.00 | 19 50.00 | 38 100.00 |  |  |  |
| Abnormal bristles | Non remarkable | 8 36.36 50.00 72.73 | 8 36.36 50.00 72.73 | 16 72.73 | 1.0000 | | |
|  | Abnormal bristles | 3 13.64 50.00 27.27 | 3 13.64 50.00 27.27 | 6 27.27 |  |  |  |
|  | Total | 11 50.00 | 11 50.00 | 22 100.00 |  |  |  |
| Skin injuries | Non remarkable | 9 42.86 50.00 90.00 | 9 42.86 50.00 81.82 | 18 85.71 |  | 1.0000 | |
|  | Skin injuries | 1 4.76 33.33 10.00 | 2 9.52 66.67 18.18 | 3 14.29 |  |  |  |
|  | Total | 10 47.62 | 11 52.38 | 21 100.00 |  |  |  |
| Tail lesion | Non remarkable | 4 16.00 44.44 33.33 | 5 20.00 55.56 38.46 | 9 36.00 |  | **1.0000** | |
|  | Lesion | 8 32.00 50.00 66.67 | 8 32.00 50.00 61.54 | 16 64.00 |  |  |  |
|  | Total | 12 48.00 | 13 52.00 | 25 100.00 |  |  |  |
| Loss of substance (tail) | Without loss | 1 6.25 50.00 12.50 | 1 6.25 50.00 12.50 | 2 12.50 | 0.0000 | 1.000 | |
|  | Small to medium loss | 3 18.75 50.00 37.50 | 3 18.75 50.00 37.50 | 6 37.50 |  |  |  |
|  | High loss | 4 25.00 50.00 50.00 | 4 25.00 50.00 50.00 | 8 50.00 |  |  |  |
|  | Total | 8 50.00 | 8 50.00 | 16 100.00 |  |  |  |
| Phase of lameness | Only when moving | 0 0.00 0.00 0.00 | 1 2.78 100.00 5.00 | 1 2.78 | 1.0421 | | 0.594 |
|  | When moving and resting | 8 22.22 50.00 50.00 | 8 22.22 50.00 40.00 | 16 44.44 |  |  |  |
|  | No weight bearing on the limb | 8 22.22 42.11 50.00 | 11 30.56 57.89 55.00 | 19 52.78 |  |  |  |
|  | Total | 16 44.44 | 20 55.56 | 36 100.00 |  |  |  |
| Joint affected | Non remarkable | 1 2.50 50.00 5.00 | 1 2.50 50.00 5.00 | 2 5.00 | 1.0000 | | |
|  | Increased size / increased filling | 19 47.50 50.00 95.00 | 19 47.50 50.00 95.00 | 38 95.00 |  |  |  |
|  | Total | 20 50.00 | 20 50.00 | 40 100.00 |  |  |  |
| Number of affected joints | Non remarkable | 6 14.29 66.67 28.57 | 3 7.14 33.33 14.29 | 9 21.43 | 1.7582 | 0.4151 | |
|  | One limb | 11 26.19 42.31 52.38 | 15 35.71 57.69 71.43 | 26 61.90 |  |  |  |
|  | More than one limb | 4 9.52 57.14 19.05 | 3 7.14 42.86 14.29 | 7 16.67 |  |  |  |
|  | Total | 21 50.00 | 21 50.00 | 42 100.00 |  |  |  |
| Difficulty rising | Non remarkable | 2 6.90 25.00 15.38 | 6 20.69 75.00 37.50 | 8 27.59 | 0.238 | | |
|  | Difficult | 11 37.93 52.38 84.62 | 10 34.48 47.62 62.50 | 21 72.41 |  |  |  |
|  | Total | 13 44.83 | 16 55.17 | 29 100.00 |  |  |  |
